# Modelling the large-scale yellow fever outbreak in Luanda, Angola, and the impact of vaccination

**DOI:** 10.1371/journal.pntd.0006158

**Published:** 2018-01-16

**Authors:** Shi Zhao, Lewi Stone, Daozhou Gao, Daihai He

**Affiliations:** 1 Department of Applied Mathematics, Hong Kong Polytechnic University, Hong Kong, China; 2 School of Mathematical and Geospatial Sciences, RMIT University, Melbourne, Australia; 3 Biomathematics Unit, Department of Zoology, Tel Aviv University, Ramat Aviv, Israel; 4 Department of Mathematics, Shanghai Normal University, Shanghai, China; University of California San Francisco, UNITED STATES

## Abstract

**Background:**

Yellow fever (YF), transmitted via bites of infected mosquitoes, is a life-threatening viral disease endemic to tropical and subtropical regions of Africa and South America. YF has largely been controlled by widespread national vaccination campaigns. Nevertheless, between December 2015 and August 2016, YF resurged in Angola, quickly spread and became the largest YF outbreak for the last 30 years. Recently, YF resurged again in Brazil (December 2016). Thus, there is an urgent need to gain better understanding of the transmission pattern of YF.

**Model:**

The present study provides a refined mathematical model, combined with modern likelihood-based statistical inference techniques, to assess and reconstruct important epidemiological processes underlying Angola’s YF outbreak. This includes the outbreak’s attack rate, the reproduction number ($R_{0}$), the role of the mosquito vector, the influence of climatic factors, and the unusual but noticeable appearance of two-waves in the YF outbreak. The model explores actual and hypothetical vaccination strategies, and the impacts of possible human reactive behaviors (e.g., response to media precautions).

**Findings:**

While there were 73 deaths reported over the study period, the model indicates that the vaccination campaign saved 5.1-fold more people from death and saved from illness 5.6-fold of the observed 941 cases. Delaying the availability of the vaccines further would have greatly worsened the epidemic in terms of increased cases and deaths. The analysis estimated a mean $R_{0}\approx 2.6\text{-}3.4$ and an attack rate of 0.09-0.15% (proportion of population infected) over the whole period from December 2015 to August 2016. Our estimated lower and upper bounds of $R_{0}$ are in line with previous studies. Unusually, $R_{0}$ oscillated in a manner that was “delayed” with the reported deaths. High recent number of deaths were associated (followed) with periods of relatively low disease transmission and low $R_{0}$, and vice-versa. The time-series of Luanda’s YF cases suggest the outbreak occurred in two waves, a feature that would have become far more prominent had there been no mass vaccination. The waves could possibly be due to protective reactive behavioral changes of the population affecting the mosquito population. The second wave could well be an outcome of the March-April rainfall patterns in the 2016 El Niño year by creating ideal conditions for the breeding of the mosquito vectors. The modelling framework is a powerful tool for studying future YF epidemic outbreaks, and provides a basis for future vaccination campaign evaluations.

## Author summary

An epidemic model for the transmission of yellow fever virus (YFV) in urban areas is formulated and implemented to study the 2016 yellow fever (YF) outbreak in Luanda, Angola. We explore the complex vector-host dynamics of this system taking into account mosquito abundance, vaccination and asymptomatic infections in the human population, that are generally not included in other modelling studies of YF. The model successfully fits the time series of weekly reported YF cases and deaths during the epidemic. This allows us to study the impact of the vaccination campaign in Luanda and hypothetical “delayed vaccination” scenarios. The transmission of YFV appeared to be oscillatory having a wave-like pattern in the basic reproduction number ($R_{0}\left(t\right)$). The oscillations are hypothesized to be due to human reaction to the reported deaths, as has been noted for other human infectious diseases, and the second wave also possibly due to El Niño rainfall patterns. We conclude that the lives saved due to the vaccination campaign before August 2016 should have been approximately 370 (i.e., approximately five-fold of the observed 73 deaths), and would have been far larger extrapolating beyond August 2016.

## Introduction

Yellow fever (YF) is a life-threatening viral disease endemic to tropical regions of Africa and South America. The disease is transmitted in urban areas primarily via the bites of infected female *Aedes aegypti* mosquitoes, which is also the vector of dengue, chikungunya and Zika viruses [1–3]. Rural and intermediate YF are transmitted by sylvatic and peri-domestic *Aedes* species in Africa. For those infected with YF, the disease incubates in the first 3-6 days of onset, after which there is an abrupt “period of infection” of intense viremia lasting for 3-4 days (fever, weakness, headache, nausea, muscle pain) [4]. This is followed by a period of remission in which the symptoms reduce and settle, and most infected individuals recover at this stage. Thus, some 70-85% of YF infections are asymptomatic or have at most very mild symptoms (i.e., clinically inapparent). However, 15-25% of patients relapse and move to a “period of intoxication” characterized by abdominal pain, vomiting, jaundice (yellow skin and eyes) and often culminating in death. The case-fatality-ratio (CFR) in this latter subset is understood to be approximately 20% among the general population, and 50% among hospitalized cases [4], although the CFR is well known to be highly variable, and dependent on the particular circumstances. Like Ebola, YF is classified as a viral hemorrhagic fever, although it is responsible for a 1000-fold more illness and death than Ebola [1].

In 2016, YF resurged in Angola to become the largest YF outbreak on record over the last 30 years [5]. In swift response, almost all global stocks of the YF vaccine were exhausted by April 2016. Similar to the Angolan experience, YF recently resurged in Brazil in December 2016, where it continues to expand towards the Atlantic coast in regions not previously deemed at risk (as of March 16, 2017) [2]. Thus there is an urgent need to gain a better understanding of the transmission patterns of YF. Here we develop a mathematical model to help identify the key epidemic processes behind the Angolan outbreak in 2015-16, and the impact and effectiveness of the vaccination campaign.

The first cases of YF in Angola were seen on December 5, 2015 but reported in the media only on January 20, 2016 [6]. By November 2016, the large YF epidemic of Angola and Democratic Republic of Congo, resulted in 962 confirmed infections including 393 reported deaths [2]. YF is vaccine-preventable and the vaccine can confer long-lasting immunity. The vaccine is suitable for individuals of age 9 months or older. As such, the Angolan government initiated a vaccination campaign to prevent the spread of yellow fever on the first week of February, 2016 [2, 7]. More than 10 million doses were needed for the whole country [6]. The center of the outbreak was in Angola’s capital, Luanda province. Estimates suggest that vaccination coverage of Luanda province was 38.0% at the end of January 2016, and reached 80.0% by mid-March 2016, and 93.0% by mid-June 2016 [2, 8–10].

**Fig 1:**
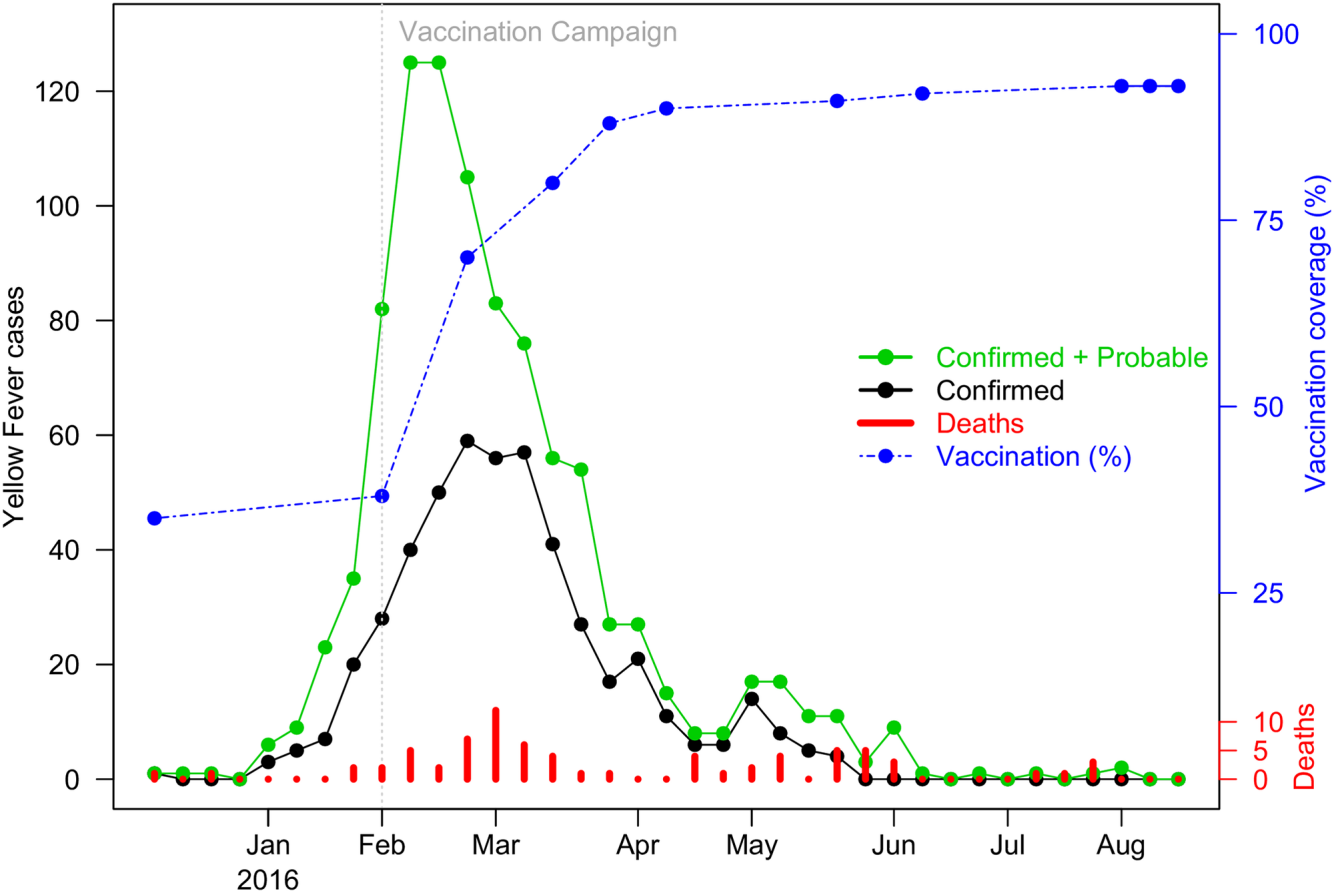
The observed yellow fever outbreak in Luanda from December 5, 2015 to August 18, 2016. Green dots denote the sum of probable and confirmed cases; black dots denote confirmed cases; red bars denote the confirmed death; and blue dots denote vaccine coverage. The vertical grey dashed line denotes the time point when the YF vaccination campaign was initiated.

Fig 1 graphs the epidemic curve of YF case numbers (probable and confirmed; as defined in Data section) in Luanda province as obtained from the WHO [8, 10]. The graph peaks in February 2016, when large-scale vaccination was introduced, and then followed by a period of rapid decline in case numbers. Despite the major vaccination effort, the epidemic proved tenacious rather than die out as predicted, and persisted for a sustained period of time forming a long “tail” in reported case numbers from April to August (see Fig 1). Also unusual is the minor peak in case numbers that occurred in May, followed soon after by an increase in deaths, despite the pressure of the vaccination and control efforts. By modelling and fitting YF time series of Luanda, our goal is to reconstruct the important epidemiological processes that help explain these different and sometimes nonintuitive features. The model allows estimation of the attack rate of the outbreak, and the basic reproduction number ($R_{0}\left(t\right)$), which was changing during the epidemic. Moreover, the model is able to explore the role of the mosquito vector, and the unusual waves of the YF outbreak, which we find would have become even more apparent had there been no vaccination. Some exploration of the role of climatic variables is also possible.

As it is well known, the basic reproduction number ($R_{0}$) is an important parameter to measure diseases’ transmissibility, and is one of the first parameters that needs to be estimated in any epidemiological study. Recall that $R_{0}$ is defined as the number of secondary cases a single typical infected individual infects over the course of its infectious period [11]. A recent study estimated $R_{0}$ to lie between 5.2-7.1 at the early stage of the 2016 YF outbreak in Angola [12]. However, $R_{0}$ was found to decrease with time as the epidemic proceeded. Kraemer *et al*. [13] estimated $R_{0}$ to be 4.8 (95% C.I.: 4.0-5.6) for Angola, although this was possibly an over-estimate given reporting rates were not stable. In summary, the literature suggests that YF is highly transmissible with direct estimates of the reproduction number being $R_{0}\approx 5$, which is almost double that of pandemic influenza ($R_{0}$ is from 1.5 to 3.6 [14–17]) and Ebola ($R_{0}$ is from 1.2 to 2.0 [18–21]). In this work, our analysis uses modern statistical inference techniques to estimate $R_{0}$ from the time-series in Fig 1. Unlike other modelling studies, our procedure also examines how reactive protective behavior (e.g., insecticide, vector-control, travel restrictions possibly in response to news and media precautions), may lead to changes in $R_{0}$, and allows us to explore the implications of this reaction.

Any model of YF must take into account that most infected individuals are asymptomatic or mild-symptomatic (individuals who show only fever but no jaundice) [3, 22–26], making the disease difficult to detect and under-reported in the first phase. With only a slight abuse of terminology, it simplifies the modelling that follows, to classify mild symptomatic individuals as asymptomatic cases. Thus asymptomatic cases refer to all individuals who do not have severe YF (i.e., without clinically apparent symptoms). It is well understood that asymptomatic YF infections can be infectious and therefore may act as “silent sources” of YFV [23]. Asymptomatic infections, thus, have the potential to play an important role in disease transmission. It was previously understood that 6 out of 7 YF infections could be asymptomatic [26]. However, a recent meta-analysis based on 11 independent studies, suggested that the asymptomatic ratio should be 55% [27]. Given the lack of information on the proportion and infectivity of asymptomatic YF cases, we examine a number of different relevant scenarios.

To the best of our knowledge, this is the first detailed modelling of YF that includes both the host and vector populations, and the asymptomatic and severe (those exhibiting fever and jaundice) cases in the host population. Previous models that assessed vaccination impact on YF have not included these fundamental components and pathways in a comprehensive approach. By fitting the time-series of the Angola outbreak, its evolution over time and its curtailment with vaccination, it becomes possible to statistically infer key model parameters. This in turn makes it possible to simulate alternative “what if” scenarios, and examine what might have happened under different vaccination schemes.

## Materials and methods

### Data and case definitions

We study time-series of YF cases from the province of Luanda of Angola with a population of 6,543,000 in 2016 [2, 8, 28]. The African Health Observatory (AHO) published weekly YF data for Luanda province reporting 941 (confirmed and probable) cases and 73 deaths over the study period from December 5, 2015 to August 18, 2016.

Probable cases (see [29]) are those “with acute onset of fever, with jaundice appearing within 14 days of onset of the first symptoms and one of the followings: (i) presence of yellow fever IgM antibody in the absence of YF immunization within 30 days before onset of illness; or (ii) positive postmortem liver histopathology; or (iii) epidemiological link to a confirmed case or an outbreak.” Confirmed cases are defined as those positive to serological or PCR testing.

Similar to the WHO [8] and Kraemer *et al*. [13], both (weekly) probable cases and confirmed cases are grouped together and are referred to simply as “YF cases” or equivalently “severe cases” in this study. YF vaccination coverage in Luanda province, obtained from AHO reports, increased from 38% on February 2, 2016 when the vaccination campaign started to 93% on August 18, 2016 (see Fig 1) [8]. The vaccination coverage was determined by a linear interpolation of reported data (see the blue dotted line in Fig 1).

### Methods

#### Yellow fever model

**Fig 2:**
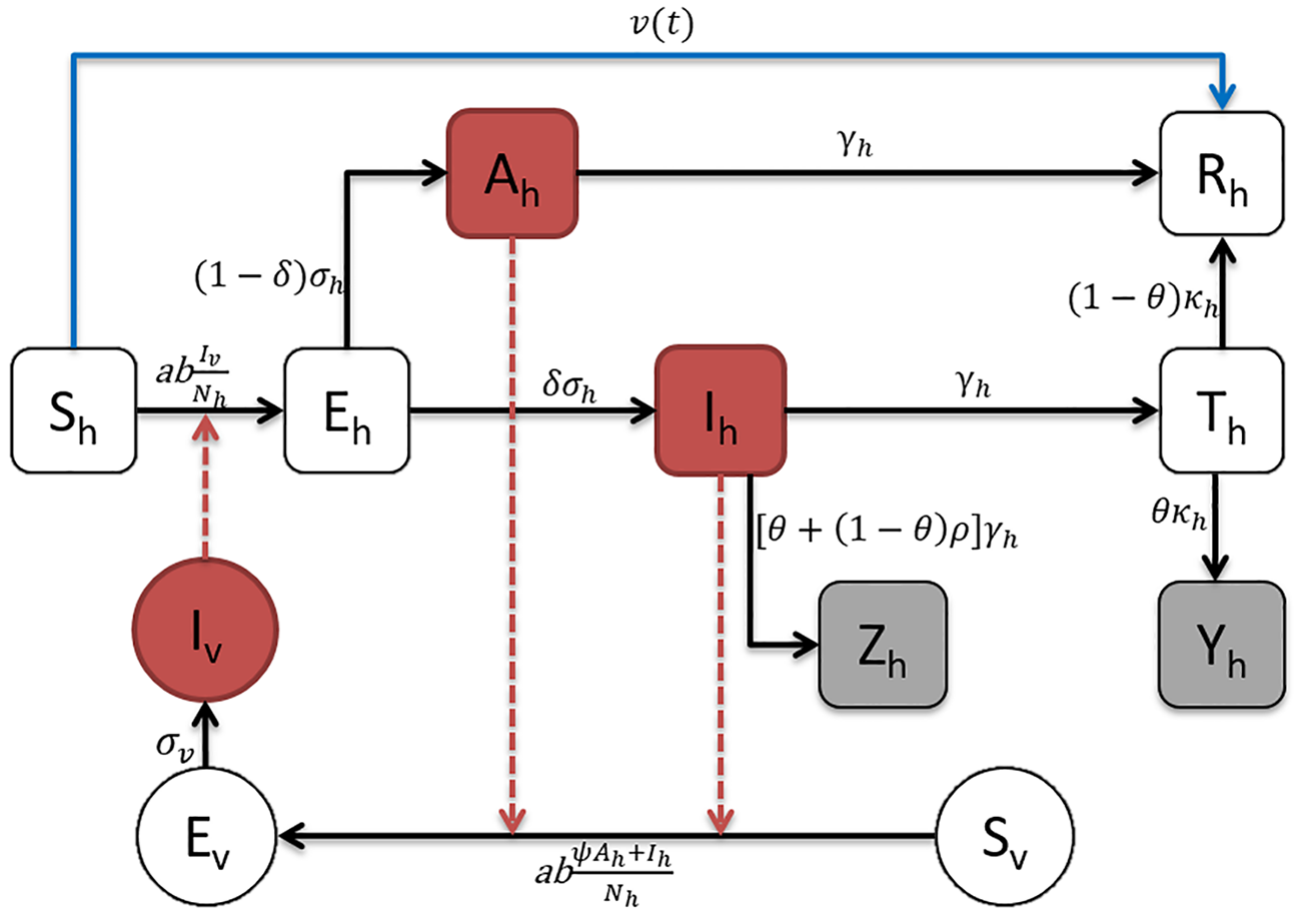
Flowchart of the yellow fever model. Black arrows represent infection status transition paths, red dashed arrows represent transmission paths and the blue arrow represents the vaccination pathway. Square compartments represent host classes and circular compartments represent vector classes. Red compartments represent infectious classes, and gray compartments are the simulated weekly reported cases (*Z*_*h*_) and deaths (*Y*_*h*_).

Since YFV is not spread by human-to-human-transmission, the standard SIR type modelling approaches (which are based solely on human-to-human transmission) are inappropriate. Instead we use a vector-host model of YFV transmission, as illustrated in Fig 2, which is based on well-known models of mosquito-borne diseases (dengue, Zika, etc.). With this vector-host model, we are able to explore the impact of different control strategies (such as vaccination, reducing mosquito abundance and human exposure to mosquito), which could not examined with approaches that fail to incorporate vector-host dynamics.

The model applied the following notations. For human host populations, *S*_*h*_ represents the number of susceptible individuals, *E*_*h*_ is the number of individuals exposed to YF but not yet infectious, *A*_*h*_ represents the asymptomatic (i.e., with clinically inapparent symptoms) cases, *I*_*h*_ the severe infectious individuals, and *T*_*h*_ the individuals in the toxic stage. Finally, *R*_*h*_ individuals have either recovered from the disease and/or have been vaccinated (or immunized by vaccination).

For the human host population, there are two main pathways, as seen in Fig 2.

H_1_) *S*_*h*_ → *E*_*h*_ → *I*_*h*_ → *T*_*h*_ → *R*_*h*_

The susceptible hosts become exposed to YFV by the bites of infectious mosquitoes, harbor the virus (move to the Exposed class) and eventually become infected (move to Infected class), enter the toxic stage (move to Toxic class) and then either eventually recover (move to Recovered class) and remain immune, or in the case of 5-50% in this stage (as specified by the CFR), die from the disease (*D*_*h*_).

H_2_) *S*_*h*_ → *E*_*h*_ → *A*_*h*_ → *R*_*h*_

In this second pathway, susceptible hosts become exposed to YF by the bites of infectious mosquitoes, harbor the virus and eventually become infected but only asymptomatic. The latter usually recover and gain future immunity.

In the above scheme, the asymptomatic (*A*_*h*_) and severe infectious (*I*_*h*_) hosts may both infect mosquitoes if they have been bitten, as shown in Fig 2. However, asymptomatic cases have a reduced transmissibility, *ψ*, when compared to that of a typical severe case. Also, note that individuals in the toxic phase no longer have viremia [1], and therefore cannot be infectious [1, 4, 30].

The vector population has only a single pathway:

V_1_) *S*_*v*_ → *E*_*v*_ → *I*_*v*_

As in usual notation, *S*_*v*_ is the number of susceptible mosquitoes. It is assumed that vertical transmission of YFV in the mosquito population is relatively small, and could reasonably be neglected for the purpose of this model [1, 31, 32]. It is also understood that mosquitoes are relatively unaffected by the mosquito-borne viruses [33].

Based on the above descriptions, we formulate an ordinary differential equations (ODEs) system that matches the scheme as illustrated in Fig 2:

$$\begin{array}{c} \left\{ \begin{array}{c} \begin{array}{cc} S_{h}^{\prime } & =-ab\frac{I_{v}}{N_{h}}S_{h}-v\left(t-t_{0}\right)\\ E_{h}^{\prime } & =ab\frac{I_{v}}{N_{h}}S_{h}-\sigma_{h}E_{h}\\ A_{h}^{\prime } & =\left(1-\delta \right)\sigma_{h}E_{h}-\gamma_{h}A_{h}\\ I_{h}^{\prime } & =\delta \sigma_{h}E_{h}-\gamma_{h}I_{h}\\ T_{h}^{\prime } & =\gamma_{h}I_{h}-\kappa_{h}T_{h}\\ R_{h}^{\prime } & =v\left(t-t_{0}\right)+\gamma_{h}A_{h}+\left(1-\theta \right)\kappa_{h}T_{h}\\ D_{h}^{\prime } & =\theta \kappa_{h}T_{h}\\ S_{v}^{\prime } & =B_{v}\left(t\right)-ac\frac{\psi A_{h}+I_{h}}{N_{h}}S_{v}-\mu_{v}S_{v}\\ E_{v}^{\prime } & =ac\frac{\psi A_{h}+I_{h}}{N_{h}}S_{v}-\sigma_{v}E_{v}-\mu_{v}E_{v}\\ I_{v}^{\prime } & =\sigma_{v}E_{v}-\mu_{v}I_{v}\\ Y_{h}^{\left(i\right)} & =\int_{\text{week}\;i}\theta \kappa_{h}T_{h}\;dt\\ Z_{h}^{\left(i\right)} & =\int_{\text{week}\;i}\left[\theta +\rho \cdot \left(1-\theta \right)\right]\cdot \gamma_{h}I_{h}\;dt \end{array} \end{array}\right. \end{array}$$ (1)

Here, *v*(*t*) represents the vaccination rate at time *t*, and *t*_0_ is the mean time period from receiving vaccination to acquiring full immunity. $Y_{h}^{\left(i\right)}$ represents the weekly recorded deaths due to YF of the *i*-th week. It is calculated as an integral which effectively sums the weekly number of toxic phase individuals (*T*_*h*_) who leave or are removed from the toxic class (*κ*_*h*_*T*_*h*_) over one week. Only a fraction (*θ*) of the latter dies, where *θ* is the CFR for severe cases. Similarly the variable $Z_{h}^{\left(i\right)}$ denotes the weekly recorded (observed) cases. This is determined through the term [*θ* + *ρ* ⋅ (1 − *θ*)] which collects the deaths (through *θ*) and the non-fatal severe cases via the severe case reporting ratio *ρ* (more detailed discussion regarding YF case counting can be found in S2 Text). It is assumed that all deaths are reported, and that the fitting procedure can estimate the reporting ratio of severe cases *ρ*.

In terms of the total host and vector population sizes (*N*_*h*_ and *N*_*v*_), the following relations must hold:

$$\begin{array}{ccc} N_{h} & = & S_{h}+E_{h}+A_{h}+I_{h}+T_{h}+R_{h}+D_{h}=\text{constant} \end{array}$$ (2)

$$\begin{array}{ccc} N_{v} & = & S_{v}+E_{v}+I_{v} \end{array}$$ (3)

In our model, *N*_*v*_ = *N*_*v*_(*t*) is time-dependent in a manner that is controlled by the mosquito birth rate *B*_*v*_(*t*) and death rate *μ*_*v*_(*t*), namely $N_{v}^{\prime }=B_{v}\left(t\right)-\mu_{v}\left(t\right)\cdot N_{v}$. Following [34, 35], we suppose that

$$\begin{array}{c} N_{v}\left(t\right)=m\left(t\right)\cdot N_{h} \end{array}$$ (4)

Here the constant *N*_*h*_ = 6,543,000 is the number of humans in Luanda province. The parameter *m*(*t*) is the time-dependent ratio of the mosquito-to-human populations that needs to be estimated. It is assumed that *m*(*t*) is an exponential cubic spline function of time with number of nodes *n*_*m*_ (see Supplementary Information section S1 Text). Nodes are distributed uniformly over the time-domain with values (*m*_*i*_) that are estimated but restricted to lie between 0 and 20. The range was chosen to reflect reality (where *m* = 20 implies $R_{0}=10$, which is beyond the upper bound of $R_{0}$ for YF).

The procedure for modelling human reactive behaviour also relies on the time-dependent parameter *m*(*t*). In practice, vector control efforts (i.e., “mosquito-fogging”) were case-driven, and fogging was implemented in localities where cases and mortality were reported. Hence it is somewhat reasonable to link mortality to the mosquito-abundance and the transmission rate (see a discussion of this in S8 Text). For a situation in which *m*(*t*) is controlled by death rates only, the cubic spline function for *m*(*t*) (Eq 4) can be replaced with a simple function based on the YF mortality (S5 Text):

$$\begin{array}{c} m\left(t\right)=m_{\text{base}}+k\cdot \exp[-D_{h}\left(t-t_{\text{lag}}\right)]. \end{array}$$ (5)

Here *m*_base_ is a constant term, *k* is a parameter controlling the strength of the death-induced human reaction, *D*_*h*_(*t*) is the yellow fever deaths of week *t* and *t*_lag_ is the lag time for the population response in reaction to mortality levels.

#### Model parameters

The model is parameterized from prior knowledge of YF, and uses parameter values that are accepted in the literature. Table 1 summarises all model parameters and their ranges. Table 2 summarises parameter values for the different scenarios and model estimates (discussed below).

**Table 1:** Summary of parameters.

Parameter	Notation	Value/Range	Unit/Remark	Source
mosquito biting rate	*a*	0.3–1.0	per vector⋅day	[36]
transmission probability from vector to host	*b*	0.10–0.75	per bite	[36]
transmission probability from host to vector	*c*	0.30–0.75	per bite	[37]
vaccination rate	*v*	0–0.043	per day	[2, 8]
host latent period	$\sigma_{h}^{-1}$	3–6	days	[2, 3, 38]
vector latent period	$\sigma_{v}^{-1}$	8–12	days	[4]
non-severe case relative infectivity	*ψ*	0.1–0.5	Nil	-
host infectious period	$\gamma_{h}^{-1}$	3–4	days	[2, 3]
severe case proportion	*δ*	15%	Nil	[3, 27]
severe case CFR	*θ*	0%–50%	Nil	[2–4, 39]
toxic phase duration	$\kappa_{h}^{-1}$	7–10	days	[2, 3]
vector lifespan	$\mu_{v}^{-1}$	4–35	days	[36, 37]
severe case reporting ratio	*ρ*	0.01–0.99	Nil	-
initial susceptible host	*S*_*h*_.0/*N*_*h*_	0.62	fixed, Nil	[8]

**Table 2:** Parameter summary for two scenarios. *X*.0 denotes *X*(*t* = 0), which is the number individuals in *X* class at the beginning of the study period.

Parameters	Notation	weak	strong	Type
mosquito biting rate	*a* (per day)	0.5	0.5	fixed
transition probability from vector to host	*b* (per bite)	0.4	0.4	fixed
transition probability from host to vector	*c* (per bite)	0.5	0.5	fixed
host latent period	$\sigma_{h}^{-1}$ (days)	4	4	fixed
host infectious period	$\gamma_{h}^{-1}$ (days)	4	4	fixed
toxic case duration	$\kappa_{h}^{-1}$ (days)	8	8	fixed
vector latent period	$\sigma_{v}^{-1}$ (days)	10	10	fixed
vector lifespan	$\mu_{v}^{-1}$ (days)	20	20	fixed
severe case proportion	*δ*	0.15	0.15	fixed
non-severe case relative infectivity	*ψ*	0.1	0.5	fixed
severe case CFR	*θ*	0.06	0.06	fixed
number of nodes	*n*_*m*_	7	7	estimated
severe case reporting ratio	*ρ*	0.71	0.72	estimated
mean *m*(*t*)	〈*m*(*t*)〉	6.34	2.79	estimated
over-dispersion	*τ*	0.002	0.0045	estimated
initial susceptible host	*S*_*h*_.0/*N*_*h*_	0.62	0.62	fixed
initial exposed host	*E*_*h*_.0/*N*_*h*_	3e-07	3.2e-07	estimated
initial non-severe host	*A*_*h*_.0/*N*_*h*_	3e-07	3.2e-07	estimated
initial severe host	*I*_*h*_.0/*N*_*h*_	3e-07	3.2e-07	estimated
initial toxic host	*T*_*h*_.0/*N*_*h*_	3e-07	3.2e-07	estimated
initial recovered host	*R*_*h*_.0/*N*_*h*_	0.38	0.38	fixed
initial susceptible mosquito	*S*_*v*_.0/*N*_*h*_	11.66	3.62	estimated
initial exposed mosquito	*E*_*v*_.0/*N*_*h*_	1.66e-06	1.93e-06	estimated
initial infectious mosquito	*I*_*v*_.0/*N*_*h*_	1.66e-06	1.93e-06	estimated
mean basic reproductive number	$\langle R_{0}\rangle$	3.41	2.57	estimated
infection attack rate (%)	AR	0.09	0.15	estimated
maximum log likelihood	MLL	-166.48	-165.4	estimated
Bayesian Information Criterion	BIC	388.9	386.75	estimated

With regard to parameters for the host population in Eq 1, $\sigma_{h}^{-1}$ and $\gamma_{h}^{-1}$ represent the host latent and infectious period respectively, with both being approximately 4 days. The latent period also indirectly allows a four-day reporting delay. Symptoms appear when patients leave the latent class, but are reported only when they leave the infectious class which is a four-day period. The toxic phase duration $\kappa_{h}^{-1}$ is set to eight days.

The parameters on mosquitoes were taken from the dengue literature, where mosquito dynamics is also modelled. Since dengue and YF viruses belong to the same family of viruses (i.e., *flaviviridae*) and share the same type of vectors (i.e., *Aedes aegypti*), we follow the practice of previous studies and assume they have similar parameter values. Specifically, parameter values for the mosquito biting rate (*a*), the transmission probabilities (*b*, *c*), and the mosqutio lifespan ($\mu_{v}^{-1}$) were taken from Massad *et al*. [40], as indicated in Table 1. The vector latent period $\sigma_{v}^{-1}$ and lifespan $\mu_{v}^{-1}$ were taken as 10 and 20 days respectively.

#### Vaccination

The term *v*(*t*) appearing in the equation for susceptible host dynamics (Eq 1), represents the time-dependent vaccination rate of the host population (blue arrow in Fig 2). It is determined by considering the equation for susceptible dynamics (from Eq 1): *dS*_*h*_/*dt* = −*abI*_*v*_/*N*_*h*_ − *v*(*t* − *t*_0_), thus, the rate of vaccinating people is *v*(*t*) and the total number of people who would normally be vaccinated by time *t* is $V\left(t\right)=\int_{0}^{t}v\left(x\right)dx+V_{0}$. Note that *t*_0_ is the mean time taken for an individual to gain full immunity after being vaccinated. Averaging the data reported by WHO and CDC [2, 3] gives *t*_0_ = 20 days. The overall cumulative vaccination for Luanda province, as reported by WHO, is plotted in Fig 1 as a percentage of the total population *N*_*h*_. That is, the y-axis plots *V*(*t*)/*N*_*h*_ × 100. Using this graph and the relation *dV*(*t*)/*dt* = *v*(*t*) allows us to reconstruct *v*(*t*) which is used in the numerical integration of Eq 1.

The constant *V*_0_ denotes pre-existing immunity of the population at the beginning of the 2015-16 YF outbreak. As the attack rate of YF is typically very small and no major outbreaks occurred in Angola since 1988, we suppose that previously built-up immunity is relatively small and acquired immunity waned significantly over the next 27 years. Nevertheless, it was assumed that at the beginning of each simulation, *V*_0_ = 38% of the population is already vaccinated to be consistent with WHO estimates. [8]. The 38% includes both the outcome of EPI (WHO’s Expanded Program on Immunization) vaccination and immunity remaining from the mass campaign in 1988 (this is incorporated by setting *S*_*h*_.0/*N*_*h*_ = 0.62 as in Table 1). Wu *et al*. [12] assumed that initial immunity was equivalent to 28% vaccination coverage, which under-estimates the WHO data. Results and Discussion section provides more information about YF vaccination doses.

The approach for modelling vaccination is adapted from our previous work on influenza [41], and avoids the need for inclusion of a separate vaccinated compartment in the model which would result in unnecessary additional complexity.

Different YF vaccination intervention scenarios are compared in order to evaluate the effectiveness of the actual national vaccination campaign. The best-fitting model to the data and the actual vaccination coverage will be taken as the “baseline scenario” as experienced in Luanda, which was initiated on February 2, 2016. This will be compared to three other additional hypothetical intervention scenarios.

- Actual vaccination campaign as experienced in Luanda (baseline scenario);
- 60, 120 and ≥ 180 days delay of vaccination campaign (hypothetical intervention scenarios).

The total observed cases as well as the total deaths are evaluated by the model for each vaccination scenario.

The 180-day delay period in fact represents a “no-vaccination” scenario. When taking into account the extra 20 days required for vaccination to be effective, anyone vaccinated 180 days after the February 2, 2016, will not gain any protective effect from the vaccination given the observation study period is only 200 days. Thus, any scenario with a delay greater than 180 days is equivalent to a no-vaccination scenario.

#### Fatality per infection and case-fatality-ratio

Monath *et al*. [4] estimated the fatality per infection of YF for the whole population to be in the range of 3-7.5% [4], and are variable across time and location. In an earlier study, Monath *et al*. estimated the fatality per infection to be in the range of 1-15% for Nigerian villages [42]. For severe YF cases, the Case-Fatality-Ratio (CFR) resulting in death (*θ*) is 20-50% or higher [4, 43–45], although the CFR is well known to be highly variable, and dependent on particular circumstances.

Given the large proportion of infected but asymptomatic YF cases, the accurate fatality per infection in Luanda cannot be determined without a comprehensive serological study, which to our knowledge has not been undertaken. But the Cases-Fatality Ratio (CFR, cases refer to severe cases) can be immediately approximated as the ratio of confirmed deaths to the confirmed (and probable) cases. For the data of Luanda province, the CFR is approximately 7.76%, and thus substantially lower than 20-50%. (Similar low estimates were noted by the WHO reports throughout the epidemic.) Moreover, because of reporting errors, we would expect the CFR to be even lower than this empirical estimate.

In our study, we choose CFR = 6% for our main simulations. But we have also carefully explored other possibilities. For example, in S2 Text, we run our fitting procedure to actually estimate the CFR given the data and find CFR = 4%. We also discuss what might be expected if the mortality data is under-reported (see S7 Text). More data and research is needed to gain a better understanding of the CFR and to check whether and how it changes over the study period.

Finally, we note that in our model, the fatality per infection is given by *δ* ⋅ *θ*, where the CFR = *θ*, while *δ* is the proportion of severe cases. In this work, we consider either fixing the CFR to a value considered realistic, or inferring the CFR from the data itself, as an extra parameter.

#### Asymptomatic infections

As reported by the CDC, “*asymptomatic or clinically unapparent infection is believed to occur in most YFV infections*” [3]. A case is defined as an asymptomatic infection only if it is confirmed strictly to have no symptoms, but is nevertheless found infectious as confirmed by RNA or serological tests. It was previously believed that 6 out of 7 YF infections could be asymptomatic [26]. Recently, a meta-analysis based on 11 studies suggested that the asymptomatic ratio should be 55% and mild cases 33% (without jaundice), the rest 12% are severe cases [27]. As mentioned, to simplify presentation all mild cases (without jaundice) were considered to belong to the asymptomatic class. Two scenarios were examined:

1. 85% asymptomatic (*δ* = 15%) and weak infectivity (*ψ* = 0.1)
2. 85% asymptomatic (*δ* = 15%) and strong infectivity (*ψ* = 0.5)

The proportion of 85% for asymptomatic infections based on [27]. Scenarios 1 & 2 differ only in terms of their weak (*ψ* = 0.1) or strong (*ψ* = 0.5) infectivity. The results for scenarios 1 & 2 (with fixed CFR = 6%) are presented in the main text, while results of flexible CFR can be found in S2 Text.

#### Basic reproduction number

Following the next generation matrix method [46, 47], the basic reproduction number for the above model equations (see Eq 1) was calculated as:

$$\begin{array}{c} R_{0}=\sqrt{\left[\psi \cdot \left(1-\delta \right)+\delta \right]\cdot \frac{a^{2}bcm}{\gamma_{h}}\cdot \frac{\sigma_{v}}{\mu_{v}\left(\sigma_{v}+\mu_{v}\right)}} \end{array}$$ (6)

as derived in S3 Text. $R_{0}\left(t\right)$ is calculated as a function of time, by assuming the mosquito-to-human population ratio to be *m* = *m*(*t*) (see Eq 4), which varies with time in a manner that may be estimated by the model fitting procedures.

**Fig 3:**
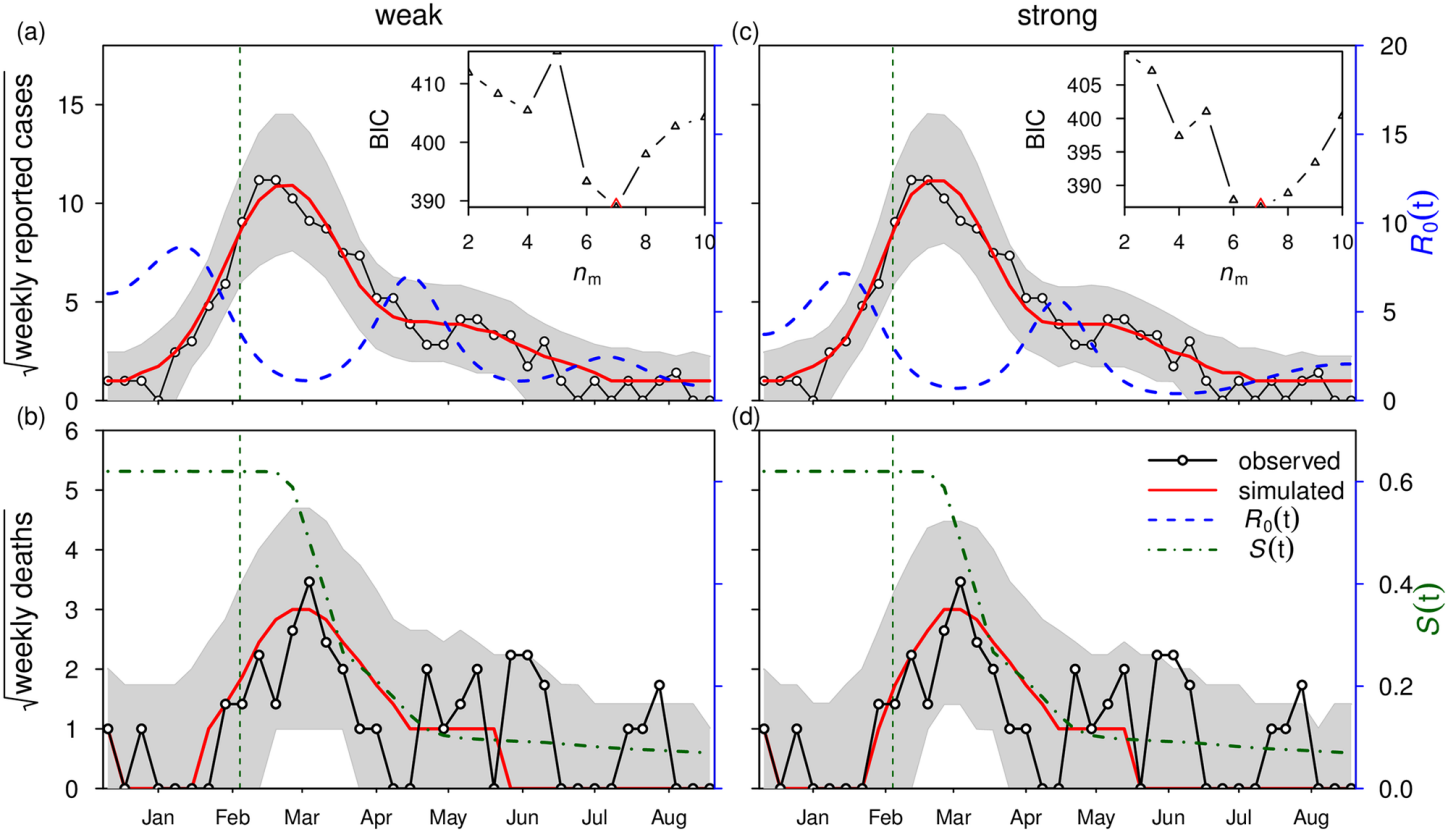
Model fitting results under two scenarios: Scenario 1 (weak infectivity with *ψ* = 0.1) in panels (a,b) and scenario 2 (strong infectivity with *ψ* = 0.5) in panels (c,d). Black line with circles denote reported cases (in (a), in the form of square-root) and reported deaths (in (b), in the form of square-root), and red line denotes model simulation median. Blue dashed line denotes the fitted basic reproduction number, $R_{0}\left(t\right)$, and the green dashed line shows the calculated host susceptible proportion, *S*(*t*) (or *S*_*h*_(*t*)/*N*_*h*_ in the model Eq 1). Shaded region represents 95% bound of 1,000 model simulations. Vertical dashed line indicates the start date of the vaccination campaign. Inset panel shows BIC as a function of the number of nodes (*n*_*m*_). The lowest BIC is attained at *n*_*m*_ = 7 in both scenarios, which is used in the main panel. Parameter values are listed in Table 2.

**Fig 4:**
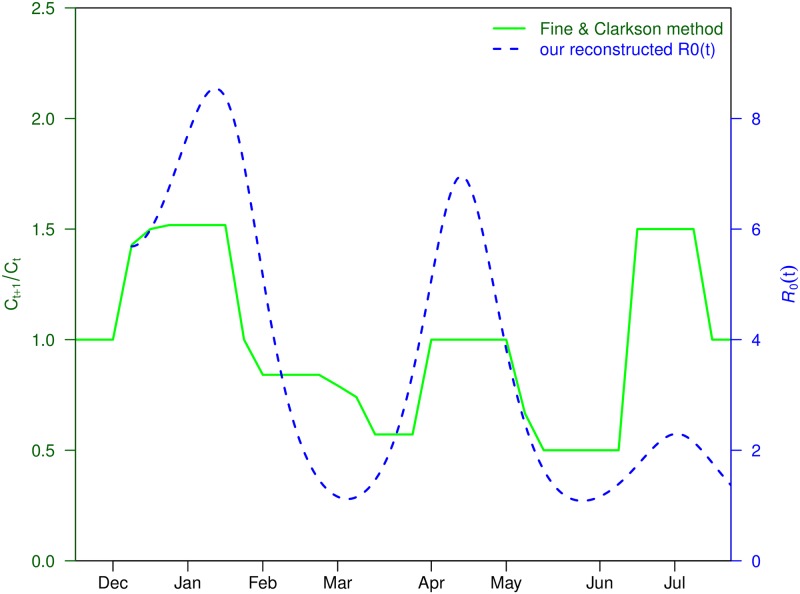
Reconstructed transmission rate *Q*(*t*) via Fine & Clakrson’s method (with a three-week shift to the left) and our estimated $R_{0}\left(t\right)$.

When the population is not fully susceptible, it is a common practice to make use of $R_{\text{eff}}$, the “effective $R_{0}$” given by $R_{\text{eff}}\left(t\right)=R_{0}\left(t\right)\cdot S_{h}\left(t\right)/N_{h}$, to describe the ability of a virus to invade the host population [48]. $R_{\text{eff}}$ incorporates both the changes in the intrinsic ability of the virus, the characteristics of the mosquito vector as well as the availability of susceptible human hosts. We note that the vaccination campaign can only reduce the availability of human susceptibles but not the intrinsic transmissibility of the virus. Vector control (e.g., mosquito fogging) can reduce the transmissibility of the virus, by reducing its vector of transmission. In Fig 3, we plot both $R_{0}\left(t\right)$ and *S*(*t*). From these, it is simple to obtain $R_{\text{eff}}\left(t\right)$. In Fig 4, we compare $R_{0}\left(t\right)$ with a reconstructed transmission rate (see next section).

### Plug-and-play inference framework

The YF outbreak in Luanda is modeled as a Partially Observed Markov Process (POMP) and makes use of the Iterated Filtering and plug-and-play likelihood-based inference frameworks to fit the data [34, 49, 50]. These are modern state-of-the-art statistical methodologies developed for fitting complex epidemiological datasets. The Maximum Likelihood Estimate (MLE) for model parameters is calculated using R package “POMP” [51].

Bayesian Information Criterion (BIC) is employed as a criterion for model comparison, and quantifies the tradeoff between the goodness-of-fit of a model and its complexity [52]. The simulations made use of the Euler-multinomial integration method with the time-step fixed to be one day [49, 53].

The model is first fitted to the observed YF cases and deaths, given knowledge of the true vaccination coverage. The mosquito abundance is assumed to be unknown but time-dependent, and is reconstructed. We allow the basic reproduction number of our model to be time-dependent, given that the mosquito abundance is not fixed and human behavior can impact $R_{0}\left(t\right)$ and change over the study period.

The parameter fitting and inference process are carefully checked, thereby giving high confidence that the fits of the observed time-series are accurate for reasons that are consistent with the true underlying epidemiological processes rather than artificial model over-fitting. We conducted tests to find the best-fit model. For each asymptomatic scenario, we studied 10 different values of *n*_*m*_ (degrees of freedom in the *m*(*t*)), and compared them with BIC. BIC quantifies the trade off of the goodness of fitting of the model and the complexity of the model—penalizing models with more variables. A smaller BIC implies a better-fit model. For the best-fit model, the profile of maximum log likelihood was calculated as a function of the reporting ratio (see S2 Text in SI for further details). The profile found is always a reasonably smooth function. The model was run 1,000 times with the estimated parameters, and the median of the model simulation matched the reported weekly cases. Therefore, we can be confident that the maximization of model’s log likelihood converged and the estimation is consistent.

The simulated weekly reported cases *Z*_*t*_ are modelled by Eq 1. The corresponding weekly observed cases, *C*_*t*_, as given by the WHO, are assumed to follow a Negative-Binomial (NB) distribution as

$$\begin{array}{c} C_{t}∼\text{NB}(n=\frac{1}{\tau },p=\frac{1}{1+\tau Z_{t}})\;\text{with}\;\text{mean}\;:\;\mu_{t}=Z_{t}, \end{array}$$ (7)

where *τ* denotes an over-dispersion parameter that needs to be estimated.

The weekly observed deaths, *D*_*t*_, and the corresponding weekly simulated deaths, *Y*_*t*_, are similarly related. Finally, the overall log-likelihood function, *l*, is given by

$$\begin{array}{c} l\left(\Theta |C_{1},\ldots ,C_{N};D_{1},\ldots ,D_{N}\right)=\sum_{t=1}^{T}\text{ln}\;[L_{t}^{\left(C\right)}\cdot L_{t}^{\left(D\right)}], \end{array}$$ (8)

where Θ denotes the parameter vector under estimation, and $L_{t}^{\left(C\right)}$ and $L_{t}^{\left(D\right)}$ are the probability measurement functions associated with *C*_*t*_ vs. *Z*_*t*_, and *D*_*t*_ vs. *Y*_*t*_, respectively. *T* denotes the total number of weeks during the study period.

The confidence intervals (C.I.) of parameters are estimated based on parameters ranges in Table 1, using the method of profile likelihood confidence intervals [35, 49]. This is demonstrated in S2 Text for the severe case reporting rate *ρ*. Parameter estimation and statistical analysis are conducted using R (version 3.3.3).

### Sensitivity analysis

The Partial Rank Correlation Coefficients (PRCCs) are adopted for the model’s sensitivity analysis [34]. Firstly, 1,000 random samples are taken for each model parameter from uniform distributions with parameter ranges as set out in Table 1. After that, for every random parameter sample set, the YF model was simulated to obtain the target biological quantities (e.g., $R_{0}$ and total number of deaths in this study). Finally, PRCCs were calculated between each parameter and target biological quantities.

## Results and discussion

### Model fitting

The results for the best-fitting model under the two scenarios (i.e., weak and strong infectivity scenarios) are shown in Fig 3. The model simulation median (of 1,000 simulations) of YF cases in Luanda is plotted in red and matches well the observed patterns seen in weekly reported cases, both before and after the national vaccination campaign. The two scenarios (for asymptomatic infectivities) both model the data with almost the same goodness-of-fit with a ΔBIC ≈ 2 (see S2 Text for the simulation results of strong infectivity scenario, i.e., scenario 2). That is, the observed and model time series are not significantly different for the two levels of infectivity [52]. As such the infectivity of asymptomatic cases cannot be accurately inferred from these data sets.

In Table 2, the over-dispersion *τ*, is notably small, which indicates the measurement model is close to a Poisson distribution (i.e., minor over-dispersion in measurement noise). This implies the reporting efforts (i.e., reporting ratios) were reasonably stable over time.

The analysis estimated a mean $R_{0}\approx 2.6\text{-}3.4$ and an attack rate of the whole period to be 0.09-0.15% (% population infected by YF) from December 2015 to August 2016. Our estimated initial and upper bound $R_{0}$ are in line with previous studies.

Asymptomatic cases were not reported, and they might be considered as a completely hidden variable. However, if the number of asymptomatic cases is very large (e.g., if the asymptomatic-to-symptomatic ratio is 6:1 or 7:1) with a weak infectivity but full immunity, this will indirectly slow down the transmission of YF in the later stages, due to herd immunity built up by these silent asymptomatic cases. If their infectivity is strong, this will increase the difficulty to control the outbreak.

**Fig 5:**
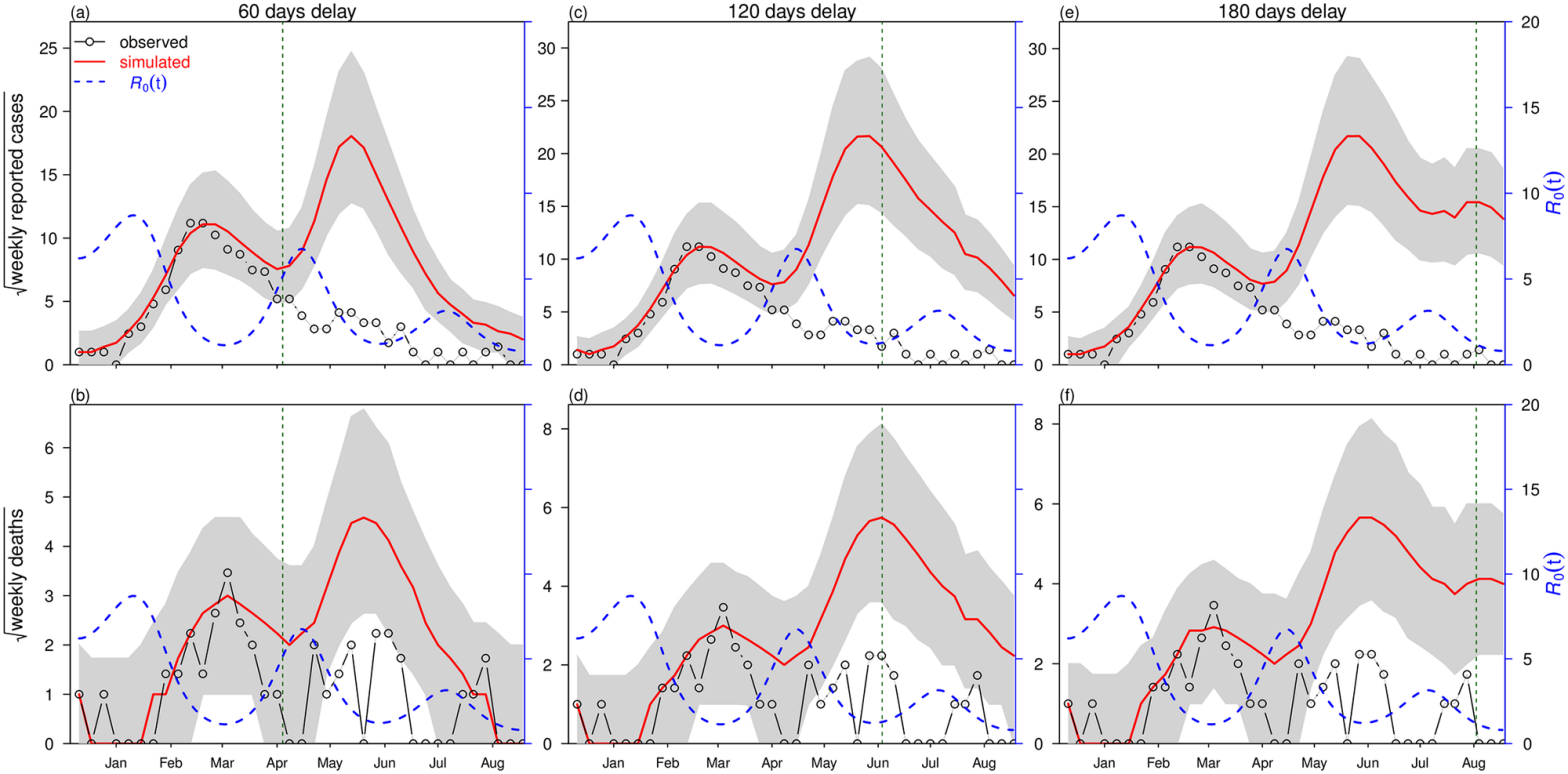
Simulation results of scenario 1 under three deferred vaccination campaign scenarios: 60 day delay in panels (a,b), 120 day delay in panels (c, d) and 180 day delay in panels (e,f). Black line with circles denote reported cases (e.g., in (a), in the form of square-root) and reported deaths (in (b), in the form of square-root), red line denotes model simulation median and blue dashed line is the fitted basic reproduction number, $R_{0}$. Shaded region represents 95% range of 1,000 simulations. The vertical dashed line represents initiation of the vaccination campaign. The number of nodes, *n*_*m*_ = 7, is adopted.

The model simulations of weekly deaths also fit the observed data well over the period of the main epidemic until the end of April 2016. While the simulated median (red line) does not predict the two relatively small and erratic peaks at the beginning of June and end of July, nevertheless they fit reasonably within the 95% bounds. (Note that similar peaks in death numbers appear in the delayed vaccination scenarios Fig 5b, 5d and 5f, where case numbers are higher. See next section.)

The observed YF deaths are relatively “noisy” compared to the continuously observed YF cases (see red bars versus green dotted line in Fig 1), which might be due to the lower case numbers involved or possibly spatial variation of the YF CFR. The same holds for individual model simulations. The total number of deaths was only 6% out of all reported cases (i.e., CFR = 6%), and 71% of the deaths appeared during the first wave. Although we cannot fit the final erratic mortality waves with high accuracy, our estimate of the total number of deaths is still a very good approximation. As can be seen in Table 3, the model’s simulated cumulative death toll matches well with the observed death toll.

**Table 3:** Impacts of vaccination campaign delay under scenario 1: Weak infectivity.

Scenario	Total reported cases	Total deaths
Observed	941	73
Baseline model	1026 [540, 1797]	77 [35, 139]
60 days delay	3143 [1604, 5584]	233 [119, 411]
120 days delay	5450 [2751, 9611]	400 [203, 724]
180 days delay	6242 [3139, 10919]	444 [226, 787]

Parameter estimates including the basic reproduction number $R_{0}$, mean mosquito-to-human ratio 〈*m*(*t*)〉, and disease attack rate are listed in Table 2. Our estimated mean $R_{0}\approx 3.0$ with excursions to $R_{0}\approx 6.0$ matches well other studies in the literature (see Introduction). The very low attack rate is an outcome of the prompt and efficient control measures by the Angolan government [2, 6, 8].

The estimated mean mosquito-to-human ratio, 〈*m*(*t*)〉, is in line with previous work by Gao *et al*. (see [54]). The estimated reporting ratio for severe cases, *ρ* = 70%, is reasonable, given the easily recognizable symptoms (jaundice), and the control effort by the government, which managed to push the vaccination coverage to more than 90% of its population within a very short period of time.

Association between the spread of YF and local climatic factors has been discussed frequently in previous studies [55, 56]. As such, we explored the possibility that local temperature and rainfall are potential factors that consistently influence the long-term transmission dynamics. Temperature was found to have no significant effect while the effects of rainfall were significant but quantitatively mild in a first analysis. The possible reasons could be: i) local precipitation is relatively minor during the study period but concentrated in March, and the weather is continuously hot and dry; and ii) duration of the outbreak is short and other factors (control measures and human reaction) played a more prominent role. Nevertheless, in what follows, we consider the possibility that the March El Niño rainfall patterns played an important role which is difficult to untangle from our analysis. A discussion is given in S6 Text.

#### Oscillations in $R_{0}\left(t\right)$ and human reactive behaviour

For the two scenarios, the analysis revealed that $R_{0}\left(t\right)$ oscillated in the interval roughly [1.0,8.5] over the 37 weeks with mean $R_{0}\approx 3.4$ of weak infectivity, and $R_{0}\approx 2.6$ for strong infectivity. The estimated $R_{0}$ (see blue dashed line in Fig 3) is approximately from 5.0-8.5 (see CI estimate of $R_{0}\left(t\right)$ on S4 Text), which is in line with Wu *et al*.’s [12, 13] analysis of the early stage of the epidemic ($R_{0}$ is from 5.2-7.1).

We now show that the oscillation in $R_{0}\left(t\right)$ is a robust feature of the dataset based on Fine and Clarkson’s [57] effective and well known methodology. Fine and Clarkson plotted *Q*(*t*) = *C*_*t*+1_/*C*_*t*_ (i.e., next week’s reported cases divided by this week’s—as used in many classic studies of childhood infectious diseases), as a function of time *t*. This index is usually regarded as the transmissibility of human-to-human infectious diseases but can be similarly exploited for vector-borne diseases. In Fig 4, we find strong waves in *Q*(*t*) (the solid green line) that match the oscillations observed in $R_{0}\left(t\right)$ (the dashed blue line). This suggests that the waves observed in $R_{0}$ are most likely a feature embedded in the data, and thus not artificial.

A closer examination of Fig 3 helps explain the oscillations in $R_{0}\left(t\right)$ and their implications. A few weeks after the initiation of the vaccination program in February 2016, the YF epidemic is seen to curtail its rapid growth and then diminish over March. Unusually, the epidemic does not rapidly crash to extinction as the usual (SIR) epidemic curve would predict. Rather, YF cases reduced gently over the next five months in a plateau from April to September. This behavior is uncharacteristic of the standard SIR epidemic model, and appears to be an outcome of the oscillations observed in the time-series estimates of $R_{0}\left(t\right)$ (see blue dashed line in Fig 3). Thus the epidemic begins to rapidly decline and turns-around only after $R_{0}$ has reduced considerably (i.e., after the peak in $R_{0}\left(t\right)$ in January). A similar decreasing trend in $R_{0}$ over Feb-Mar 2016 was also noted by [12]. This implies that although vaccination was important for the main epidemic’s demise, the February decline in $R_{0}$ was also likely to have played an important role. Interestingly, the sustained period of cases in April (rather than the expected drop to disease extinction) occurs when $R_{0}$ increases again. Since theoretically, $R_{0}$ should remain unaffected by vaccination, we suppose the changes in $R_{0}$ are likely due to changes in other factors (e.g., control measures and behavior, or possibly weather patterns) that may influence the vector population and its transmission.

Human reaction in responding to YF deaths (i.e., the “recent-death-driven” human reaction) could also be involved in this complex wave-like behavior of $R_{0}\left(t\right)$ (see S8 Text), which could be due to the interference of multiple disease control strategies. The control efforts on mosquito-eradication increased because (or follows) the number of YF cases and deaths increased. After the national vaccination campaign began in the first week of February, 2016, and reported death decreased, the mosquito control effort may have also slowed down (possibly due to limited funding and resources). This is biologically reasonable, and could be due to protective behavioral changes such as usage of insecticide, mosquito repellent, movement restrictions, cordon sanitaire and general vector control. Thus, we hypothesize that the human behaviour (e.g., usage of mosquito insecticides, repellents and nets) in response to cases and deaths were witnessed during Luanda’s YF epidemic, of which reduced disease transmission followed high mortality and vice versa. Such a process would drive the YF case numbers to follow dynamics that differ substantially from the standard SIR epidemic curve, and possibly even induce waves. Similar phenomena were reported previously for the 1918 influenza pandemic [58, 59]. Therefore, we hypothesize that human behavior could correspond to the delay from YF deaths to the transmission rate for the next YF wave.

As mentioned in the Methods section, when modelling reactive behaviour we replace the cubic spline function (with 7 nodes) for *m*(*t*) (Eq 4) with a simple function based on the YF mortality. When we do this, almost identical results are obtained (see S5 Text). This simply means that the death data are an excellent proxy for $R_{0}$ (since *m*(*t*) directly drives $R_{0}$ (see Eq 6).

#### Generation time

The generation time (GT, i.e., the time between two successive infections) is supposed to be responsible for the delay from YF transmission waves, then, to YF cases and, finally, to YF deaths. The generation time equals the sum of latent and infectious periods of the host [60], and the sum of latent and infectious periods of the mosquito. However, mosquitoes have a short lifespan and could die before the loss-of-infectiousness of YF. Precisely, the life of adult (female) mosquito includes three parts: pre-infection, latent and infectious period. In this work, we fix the mean lifespan (*L*_*v*_) of mosquito to be 20 days, and the mean latent period to be 10 days. For the pre-infection period, we use the formula of mean age at infection (for typical childhood infections): $L_{v}/\left(1+R_{0}\right)$ [48]. If we fix $R_{0}=3.0$ (see Table 2), the mean age at infection of mosquito is 5 days. Thus, the sum of latent and infection periods of mosquito will be 20 − 5 = 15 days. The sum of latent and infectious periods of human is 4 + 4 = 8 days. Thus, the GT of YF is 23 days, which explains the time delay between the maxima of $R_{0}\left(t\right)$ and the maxima of reported weekly cases. GT will be between 18 and 28 days, which is in line with [13].

### What caused the second wave in the oscillatory pattern of $R_{0}\left(t\right)$?

For most years in Luanda, the rainy season is between November-May but the most accumulation of rain occurs in March-April [61]. The year 2016 was an El Niño year and it brought dramatic and unpredictable flooding events especially in the March-April period, thereby leading to conditions ideal for growth in mosquito populations. As in 1971, YF outbreak in Luanda, local water-storage containers (mainly the larger ones) serving the community but also in most homes, accounted for 85% of the *Ae. aegypti* larval breeding sites [61, 62]. As vividly described by Moreira [63]: “*The 2016 outbreak coincided with unusually heavy rains and a severe El Niño weather pattern. We are also suffering from an economic crisis and poor sanitary conditions. All these factors created a fertile environment for an increase in the mosquito population. The outbreak reached its peak in February and has been declining since (i.e., population numbers, not* $R_{0}$). *We have much more vaccine now (in September 2016) than we had earlier in the epidemic. The response interventions are involving communities successfully. The dry season arrived in May (2016) and since then the mosquito population has diminished*.”

Thus after the peak of the YF outbreak had passed, and the vaccination program was in progress, the local March-April El Niño rains were enhancing mosquito breeding conditions. It is surprising that simultaneously one of Luanda’s largest malaria epidemics ever was underway (“*During the first quarter of 2016, the number of cases of malaria increased dramatically to 1,531,629, up from 980,192*” [64]). We suggest that these conditions may also be responsible for the unusual but robust second wave that is observed in the time series of $R_{0}\left(t\right)$.

Since we only model a single province, Luanda, and the YF transmission spread relatively rapidly throughout the province, we might assume the effects of spatial heterogeneity are likely to be minimal. However, we do not possess sufficiently detailed data to perform a careful analysis of spatial effects, and effects at the micro-scale may be important as in other diseases such as dengue [65]. Kraemer *et al*. [13] have discussed the importance of spatial effects for YF over all provinces of Angola, and there is a possibility that geographic waves generated from surrounding provinces could play some part in the appearance of multiple YF waves in Luanda province. Hence future work and more comprehensive data are needed to examine these possibilities.

### Vaccination

There are many possible ways to evaluate the effects of a delayed vaccination campaign when compared to the baseline scenario that was implemented in practice in Luanda. The approach followed here is to simply delay the exact same baseline scenario (in terms of doses per week) by a fixed time interval until the end of the observation period arrives. It is difficult to extend beyond the observation period without introducing an unacceptable rate of errors. This can be seen in the large confidence intervals for $R_{0}$ towards the end of the observation period (see S4 Text).

The results of 60, 120, and 180 days delay of the vaccination campaign for the 2016 yellow fever outbreak are presented in Fig 5 for the scenario 1 (*ψ* = 0.1). The total reported cases and total deaths are calculated for four vaccination scenarios (including the baseline) and outcomes are listed in Table 3. The 180-day delay is included, because it gives an impression of what might happen when vaccination is unavailable, as mentioned.

The baseline scenario (actual vaccination or 0-delay) results in an estimated 73 deaths associated with YF in the study period, which matches the observed number. With a 60-day delay to the vaccination roll-out, YF deaths saved were 2.2-fold of the observed number (see Fig 5a and 5b). With a 120-day delay, the YF death saved were 4.5-fold of the observed number (see Fig 5c and 5d). With a 180-day delay, YF deaths saved were 5.1-fold of the observed number (see Fig 5e and 5f). The latter result is a good approximation to what might have occurred if there were no vaccination campaign in Luanda up to August 2016. All of these results show that delaying the vaccination campaign would have greatly enhanced the epidemic in terms of infectious cases and mortality. We also investigated the “vaccination delay” situation under different scenarios (see S2 Text), and found our main results of “deaths prevented” largely holds. In addition, we also considered the scenario of “what if deaths were under reported” (i.e., there was a constant proportion of YF deaths not reported, see S7 Text for details), we report our main results are also robust.

A clear feature of the simulated outcomes with delayed vaccination (red lines in Fig 5), is the noticeable second wave of YF cases and deaths that appear. This feature becomes even more prominent in a situation of no vaccination (see Fig 5e and 5f). Returning to Fig 1, we also see strong signs from the observed time series of YF in Luanda, that the outbreak may have indeed occurred over two waves. Hence, even with Luanda’s large-scale vaccination campaign, the multiple-wave feature is noticeable in the observed time series, which implies considerable fluctuations in the driving force ($R_{0}$) or other factors.

#### Vaccine usage

As discussed in the main text, we examined scenarios when an identical vaccination scheme to that observed in Luanda was delayed by 60 and 120 days. Here we show the number of doses given in these delayed schemes is almost identical to the baseline scheme varying at most by 5% (for delay of 120 days, 3.44 million doses). The 180-day delay is included because, as explained in the text, it is so late that it is actually a no-vaccination scenario (see below).

The calculations are based on Luanda having a population size of 6,543,000, with 38% of the population having immunity before the epidemic (November 2015). The baseline scenario characterized what happened in practice between December 2015 and August 2016. By August 2016, the vaccine-dose usages are summarized for all four scenarios in Table 4. The total reported observed cases as well as total deaths are evaluated by the model for each different vaccination scenario.

**Table 4:** Vaccine doses used during yellow fever outbreak (from Dec 5, 2015 to Aug 15, 2016) under four vaccination delay situations.

Delay	Vaccinated population since outbreak	Doses usage (person dose)
0 day	93% − 38% = 55%	3,598,650
60 days	91% − 38% = 53%	3,467,790
120 days	90.5% − 38% = 52.5%	3,435,075
180 days	54% − 38% = 16%	1,046,880

A 180-day delay period is considered to be a no-vaccination scenario. When taking into account the extra 20 days required for the vaccine to be effective, anyone vaccinated 180 days after December 2015, will not gain any protective effect from the vaccination given the observation study period is only 200 days. The impact of the vaccination goes beyond the study period. Anyone vaccinated in the last twenty days will change their status from susceptible to recovered after 15 August 2016 (i.e., the end of the study period).

### Sensitivity analysis

**Fig 6:**
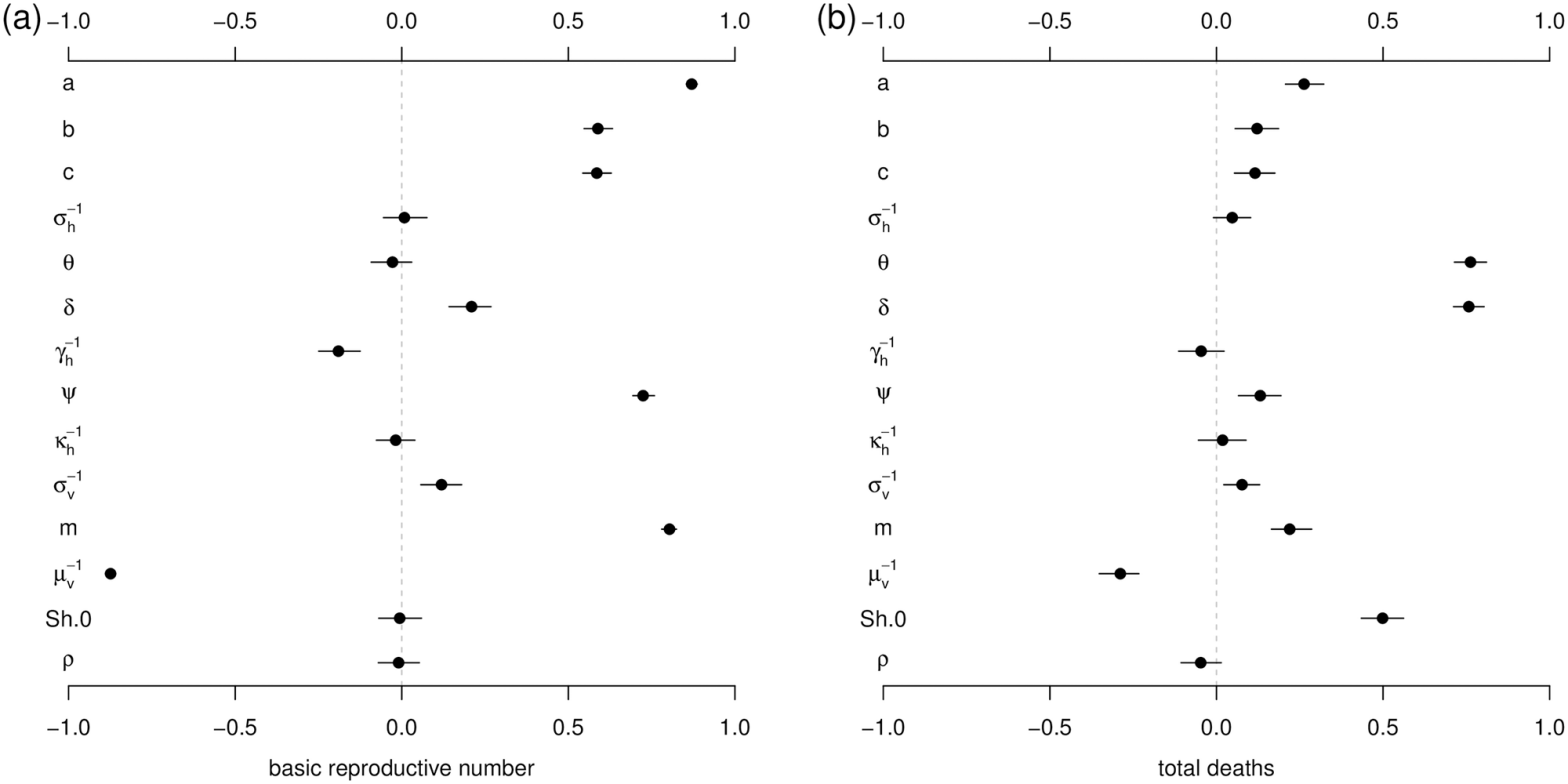
The Partial Rank Correlation Coefficients (PRCCs) of basic reproduction number (panel (a)) and total deaths (panel (b)) with respect to model parameters. *S*_*h*_.0 denotes the initial susceptible ratio (*S*_*h*_.0/*N*_*h*_). The black dots are the estimated correlations and the bars represent the 95% C.I.s. The ranges of parameters are given in Table 1.

Results of sensitivity analysis are presented in Fig 6 and indicate how model parameters impact the basic reproduction number $R_{0}$ and the death toll. $R_{0}$ is most sensitive to vector biting rate (*a*) and the vectors’ lifespan ($\mu_{v}^{-1}$), indicating the importance of the mosquitoes’ role in disease transmission. The total deaths are considerably sensitive to the proportion of severe cases (*δ*), the case-fatality rate of severe cases (*θ*) and the initial number of susceptibles (i.e., the ratio *S*_*h*_.0/*N*_*h*_).

### Conclusion

Using modern likelihood based statistical inference techniques, it was possible to fit a vector-host epidemic model successfully to the surveillance data collected for the YF outbreak in Luanda, Angola in 2016. We were thus able to assess the success of the vaccination campaign as rolled out in Luanda. While there were in reality 73 deaths reported over the 37-week study period, the model showed that the vaccination campaign saved from death 5.1-fold of observed deaths and prevented from illness 5.8-fold of observed cases, over the study period, and no doubt many more if we were to extrapolate beyond the study period. This was determined by simulating Luanda’s YF outbreak in the absence of any vaccination. The national vaccination campaign was also found to be timely, in that delaying the availability of the vaccination any further would have greatly enhanced the epidemic in terms of number of YF cases and mortality.

The change in the number of YF cases over time in Luanda suggests the possibility that the outbreak occurred in two waves over the 37-week study period. The modelling and sensitivity analysis demonstrated that this is a robust feature (see S5 Text), which would have become far more prominent had the vaccination campaign been reduced in intensity. The appearance of waves implies that $R_{0}$ must oscillate to some degree in time. Reconstruction of the underlying dynamics reveals that $R_{0}$ is strongly out of phase with mortality, so that $R_{0}$ decreases when the number of deaths increase, and vice versa. Thus we hypothesize that the high death rates and number of cases influenced Luanda’s population behavioral response which in turn led to some reduction of disease transmission during the years of high mortality. Behavioral responses may typically involve using more insecticide, mosquito repellent, insecticide-treated bednets and broader vector-control programs, as outlined in S8 Text. In Luanda, it also involved cordon sanitaire, and movement restriction with the aim of reducing transmission through the wider population [66].

Such behavioral changes are able to modulate the basic reproduction number, which in turn can lead to waves in the YF case numbers. A similar phenomenon was reported for the deadly pandemic influenza (e.g., 1918 influenza pandemic with a fatality rate (per infection) 2% and an attack rate 1/3) [58, 59] but never in mosquito-borne disease since either the CFR or the AR is typically low. Moreover, we showed how a simpler model that explicitly incorporated human behavior reproduces the observed data in Fig 1 (see S5 Text). This may be the first example of mortality-driven basic reproduction number in a mosquito-borne disease outbreak. While this possibility appears to hold in other epidemiological contexts (e.g., Spanish flu [58, 59]), it would be beneficial to check this further for vector-host systems. In the case of Luanda’s YF outbreak, $R_{0}$ is likely to have also been affected by the sporadic but heavy El Niño rainfall, which in turn could influence mosquito population numbers. Such a process could occur even if there is no visible long-term correlation between climate (rainfall) and the vector dynamics.

The modelling approach described here provides a basis for future vaccination campaign evaluations. Since the YF mortality appeared to lead to oscillations in the basic reproduction number ($R_{0}$), this possibility should be considered in the development of short-term prediction tools of the spread of YF. The general approach should be of benefit in mitigating the spread and impact of YF outbreaks in the future.

## Supporting information

S1 Text Fitting *m*(*t*) with BIC. (PDF)

S2 Text Different model scenarios. (PDF)

S3 Text Basic reproduction number. (PDF)

S4 TextEstimate the confidence interval of $R_{0}\left(t\right)$.(PDF)

S5 Text Underlying oscillation in basic reproductive number. (PDF)

S6 Text Impacts of temperature and rainfall. (PDF)

S7 Text Assuming deaths were under-reported (CFR = 15%). (PDF)

S8 Text Impact of vector control and possible impact of climate. (PDF)
