# Supplementary material for: Modelling the large-scale yellow fever outbreak in Luanda, Angola, and the impact of vaccination
Source: PLoS Negl Trop Dis. 2018 Jan 16;12(1):e0006158. doi: 10.1371/journal.pntd.0006158 (PMC5798855; doi:10.1371/journal.pntd.0006158)
Supplement: S1 Text — (PDF) [file pntd.0006158.s001.pdf]

Modelling the Large-scale Yellow Fever Outbreak in Luanda, Angola, and the Impact of Vaccination

Shi Zhao[1], Lewi Stone[2,3,*], Daozhou Gao[4] & Daihai He[1,*]

1 Department of Applied Mathematics, Hong Kong Polytechnic University, Hong Kong, China
2 School of Mathematical and Geospatial Sciences, RMIT University, Melbourne, 3000, Australia
3 Biomathematics Unit, Department of Zoology, Tel Aviv University, Ramat Aviv, Israel
4 Department of Mathematics, Shanghai Normal University, Shanghai, China

* Corresponding: D.H. daihai.he@polyu.edu.hk & L.S. lewistone100@gmail.com

S1 Fitting $m(t)$ with BIC

We performed extensive testing on fitting the model with different degrees of freedom in $m(t)$, and calculated the BIC as a function of the number of nodes in $m(t)$ from 2 to 10. This ensured that a wide range of possible shapes or profiles for $m(t)$ were explored, including possible constant, and monotonically increasing or decreasing profiles. The best model fit was chosen based on the smallest BIC, and the profile maximum log likelihood was determined as a function of the reporting ratio. The MLL profiles were plotted as function of ρ, and yielded a smooth curve, which is an indicator of convergence. We also calculated the MLL (or BIC) as function of n_m, the MLLs match. Thus we are confident that our MLL is the true maximum. the convergence of the maximization of log likelihood of the model given the data is guaranteed.

The median of 1,000 stochastic simulations of the best-fitting model matched the observed data, which indicates that it is the best model in all situations we explored.

All of the above steps were repeated (which involves fitting of dozens of models/parameter setting) under four different asymptomatic assumptions (as outlined in the main text). The possible impacts of climate and human behavioral responses (death driven vector control measure) were also considered (see main text and section S6). Thus the total computational effort is huge.

We considered using three well known test indices: AIC, BIC, DIC but ultimately decided on using BIC. This is because first, DIC has a number of known problems we prefer to avoid (see http://avansp.github.io/2014/11/02/DIC-AIC-BIC.html). Second, BIC is more appropriate than AIC in our work here, since the size of the data is relatively small. In the small sample size situation, a secondary AIC (i.e., AICc) can be considered [1]. Out investigation have made it clear that both BIC and AICc lead to the same conclusions in our examples.

References

1. Burnham KP & Anderson DR. Model selection and multimodel inference: a practical information-theoretic approach. Springer Science & Business Media. 2003.
