# Supplementary material for: Modelling the large-scale yellow fever outbreak in Luanda, Angola, and the impact of vaccination
Source: PLoS Negl Trop Dis. 2018 Jan 16;12(1):e0006158. doi: 10.1371/journal.pntd.0006158 (PMC5798855; doi:10.1371/journal.pntd.0006158)
Supplement: S2 Text — (PDF) [file pntd.0006158.s002.pdf]

Modelling the Large-scale Yellow Fever Outbreak in Luanda, Angola, and the Impact of Vaccination

Shi Zhao[1], Lewi Stone[2,3,*], Daozhou Gao[4] & Daihai He[1,*]

1 Department of Applied Mathematics, Hong Kong Polytechnic University, Hong Kong, China
2 School of Mathematical and Geospatial Sciences, RMIT University, Melbourne, 3000, Australia
3 Biomathematics Unit, Department of Zoology, Tel Aviv University, Ramat Aviv, Israel
4 Department of Mathematics, Shanghai Normal University, Shanghai, China

* Corresponding: D.H. daihai.he@polyu.edu.hk & L.S. lewistone100@gmail.com

S2 Different model scenarios

S2.1 Confidence Interval of ρ

Fig. S2 shows the maximum log likelihood as a function of reporting ratio ρ. The 95% confidence interval of ρ is (0.52, 0.95) for weak infectivity and (0.51, 0.99) for strong infectivity.

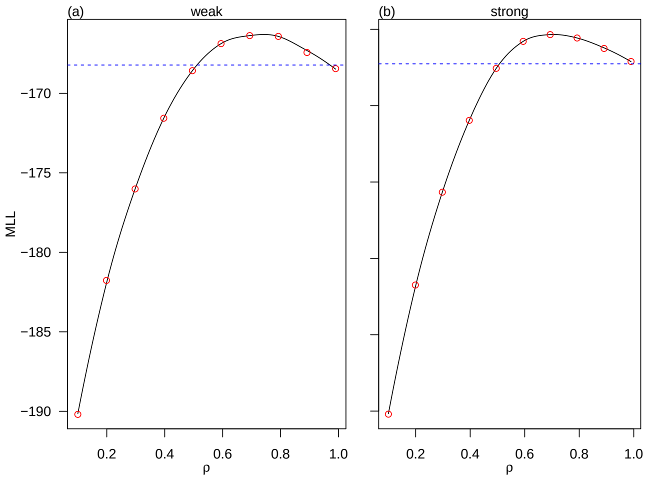

Fig S2. Maximum log likelihood (MLL) as a function of the reporting ratio ρ. Blue dashed lines are the threshold of 95% significance level ($\frac{1}{2}\chi^2_{1;95\%}$ below the max. of the MLL function). Panel (a) is under weak infectivity and panel (b) strong infectivity.

S2.2 Scenario 2: strong infectivity $\psi = 0.5$

Fitting results of scenario 2 (strong infectivity) are shown in Fig. 3 (c,d). Parameter estimates are listed in Table 2. The estimated basic reproduction number, $\mathcal{R}_0$ (see blue dashed line in Fig. S3, rises to over 8.5 at the first peak and exhibits two major waves

during the study period. The estimated reporting ratio for severe YF cases is similar as the weak infectivity scenario (see Table 2).

The results of 60, 120 and 180 days delay of the vaccination campaign are presented on Fig. S3 and Table S2.

Table S2. Impacts of vaccination campaign delay under strong infectivity scenario.

Scenario	Total reported cases	Total deaths
Observed	941	73
Baseline model	984 [540 , 1537]	80 [41 , 131]
60 days delay	3026 [1651 , 4717]	238 [128 , 378]
120 days delay	4522 [2465 , 7219]	354 [194 , 568]
180 days delay	4682 [2541 , 7312]	362 [199 , 587]

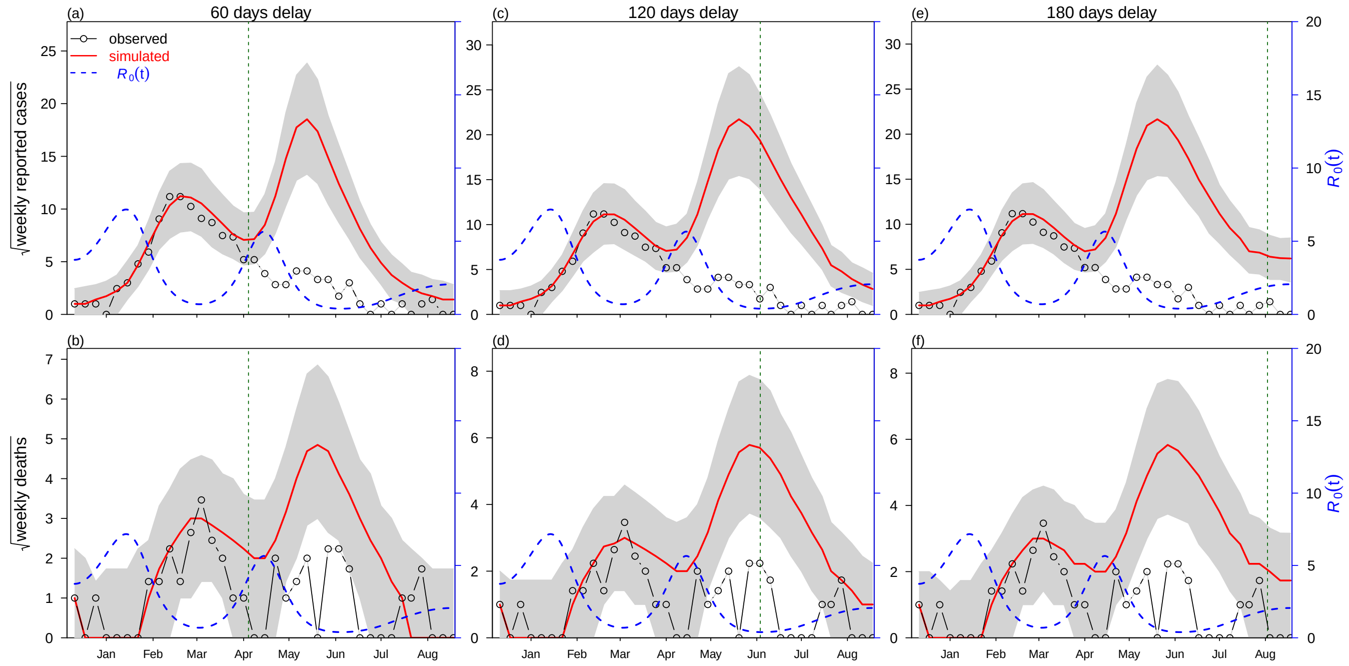

Fig S3. Simulation results (strong infectivity) under deferred vaccination campaign for 60 days in panels (a,b), 120 days in panels (c, d) and 180 days in panels (e,f). The observed cases (in the form of square-root) given by black circles, model simulation median (in the form of square-root) is in red and the fitted basic reproduction number, $\mathcal{R}_0$, is blue dashed line. Shaded region represents 95% range of 1,000 simulations. The vertical dashed line is the time point when the vaccine campaign started. The number of nodes, $n_m = 7$, is adopted.

For $\psi = 0.95$, we also examined the outcome when non-severe case relative infectivity was extremely large (or strong), namely $\psi = 0.95$. The results so obtained were very similar to results for $\psi = 0.5$. The mean $\mathcal{R}_0$ reduced to 2.47 from 2.57, and the infection attack rate increased to 0.13% from 0.12%. The deaths prevented was almost the same.

S2.3 Fitting CFR

Here we give results for fitting CFR, rather than fixing CFR to the constant $\theta = 0.06$. The model simulation results are summarized in Table S3, Table S4, Table S5, and Fig. S4, S5 and S6.

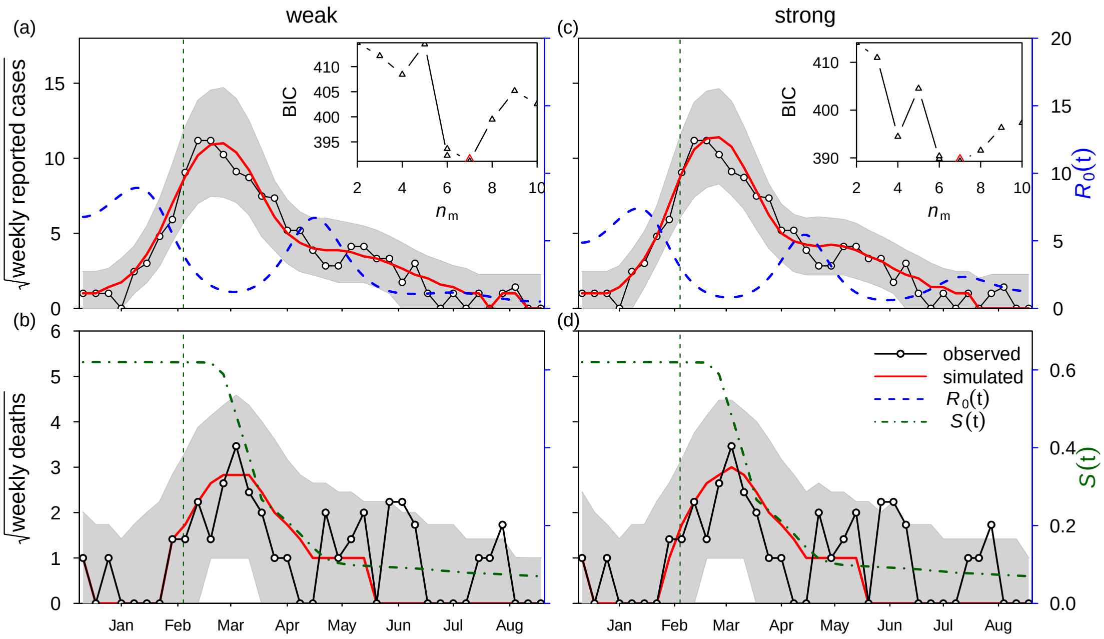

Fig S4. Model fitting results for fitting CFR with weak infectivity (a,b) and strong infectivity (c,d) of non-severe cases. Black line with circles denotes reported cases (in the form of square-root), and red line denotes model simulation median (in the form of square-root). Blue dashed line denotes the fitted basic reproduction number, $\mathcal{R}_0(t)$, and the green dashed line shows the calculated host susceptible proportion, $S(t)$. Shaded region represents 95% bound of 1,000 model simulations. The vertical dashed line indicates the starting date of the vaccination campaign. Inset panel shows the BIC as a function of the number of nodes (n_m). The lowest BIC is attained at $n_m = 7$ in both scenarios, which is used in the main panel. Parameter values are listed in Table S3.

Weak infectivity scenario With a 60-day delay to the vaccination roll-out, YF deaths saved were 2.3-fold of the observed number (see Table S4). With a 120-day delay, the YF death saved were 4.4-fold of the observed number (see Table S4). With a 180-day delay, YF deaths saved were 4.7-fold of the observed number (see Table S4).

Strong infectivity scenario With a 60-day delay to the vaccination roll-out, YF deaths saved were 2.3-fold of the observed number (see Table S5). With a 120-day delay, the YF death saved were 4.1-fold of the observed number (see Table S5). With a 180-day delay, YF deaths saved were 4.4-fold of the observed number (see Table S5).

There were differences in estimates of death prevented, but after considering the width of the 95% CI, the differences were small.

S2.4 The basic equations - reporting severe cases

Given the definition for YF reported cases corresponds to severe cases (as opposed to all infections), we seek the most natural tapping point to identify them in the equations (see Fig. 2 and Eqn. 1). We assume that to be identified as severe, they would have to reside in the infected compartment for some time. As such we have allowed case reporting to be proportional to the rate at which individuals move *into* the toxic phase ($\gamma \cdot I_h$). Note that similar results would be obtained had we let cases be proportional to

Table S3. Parameters summary for fitting CFR. $X.0$ denotes $X(t = 0)$, which is the number individuals in X class at the beginning of the study period.

Parameters	Notation	weak	strong	Type
mosquito biting rate	a (per day)	0.5	0.5	fixed
transition probability from vector to host	b	0.4	0.4	fixed
transition probability from host to vector	c	0.5	0.5	fixed
host latent period	σ_h^{-1} (days)	4	4	fixed
host infectious period	γ_h^{-1} (days)	4	4	fixed
toxic case duration	κ_h^{-1} (days)	8	8	fixed
vector latent period	σ_v^{-1} (days)	10	10	fixed
vector lifespan	μ_v^{-1} (days)	20	20	fixed
severe case proportion	δ	0.15	0.15	fixed
non-severe case relative infectivity	ψ	0.1	0.5	fixed
severe case CFR	θ	0.04	0.04	estimated
number of nodes	n_m	7	7	estimated
severe case reporting ratio	ρ	0.51	0.46	estimated
mean $m(t)$	$\langle m(t) \rangle$	6.68	3.12	estimated
over dispersion	τ	0.0047	0.0037	estimated
initial susceptible host	$S_h.0/N_h$	0.62	0.62	fixed
initial exposed host	$E_h.0/N_h$	2.9e-07	5e-07	estimated
initial non-severe host	$A_h.0/N_h$	2.9e-07	5e-07	estimated
initial severe host	$I_h.0/N_h$	2.9e-07	5e-07	estimated
initial toxic host	$T_h.0/N_h$	2.9e-07	5e-07	estimated
initial recovered host	$R_h.0/N_h$	0.38	0.38	fixed
initial susceptible mosquito	$S_v.0/N_h$	16.26	6.06	estimated
initial exposed mosquito	$E_v.0/N_h$	1.24e-06	1.33e-06	estimated
initial infectious mosquito	$I_v.0/N_h$	1.24e-06	1.33e-06	estimated
mean basic reproductive number	$\langle \mathcal{R}_0 \rangle$	3.37	2.82	estimated
infection attack rate (%)	AR	0.16	0.12	estimated
maximum log likelihood	MLL	-165.44	-164.52	estimated
Bayesian Information Criterion	BIC	391.14	389.3	estimated

Table S4. Impacts of vaccination campaign delay (fitting CFR) with weak infectivity of non-severe cases.

Scenario	Total reported cases	Total deaths
Observed	941	73
Baseline model	991 [535 , 1659]	76 [36 , 138]
60 days delay	3142 [1698 , 5255]	240 [126 , 403]
120 days delay	5287 [2839 , 8885]	396 [201 , 676]
180 days delay	5514 [2974 , 9170]	413 [223 , 709]

Table S5. Impacts of vaccination campaign delay (fitting CFR) with strong infectivity of non-severe cases.

Scenario	Total reported cases	Total deaths
Observed	941	73
Baseline model	1046 [593 , 1582]	78 [40 , 122]
60 days delay	3290 [1859 , 5030]	238 [133 , 373]
120 days delay	5162 [2910 , 7837]	372 [211 , 580]
180 days delay	5638 [3205 , 8621]	397 [224 , 602]

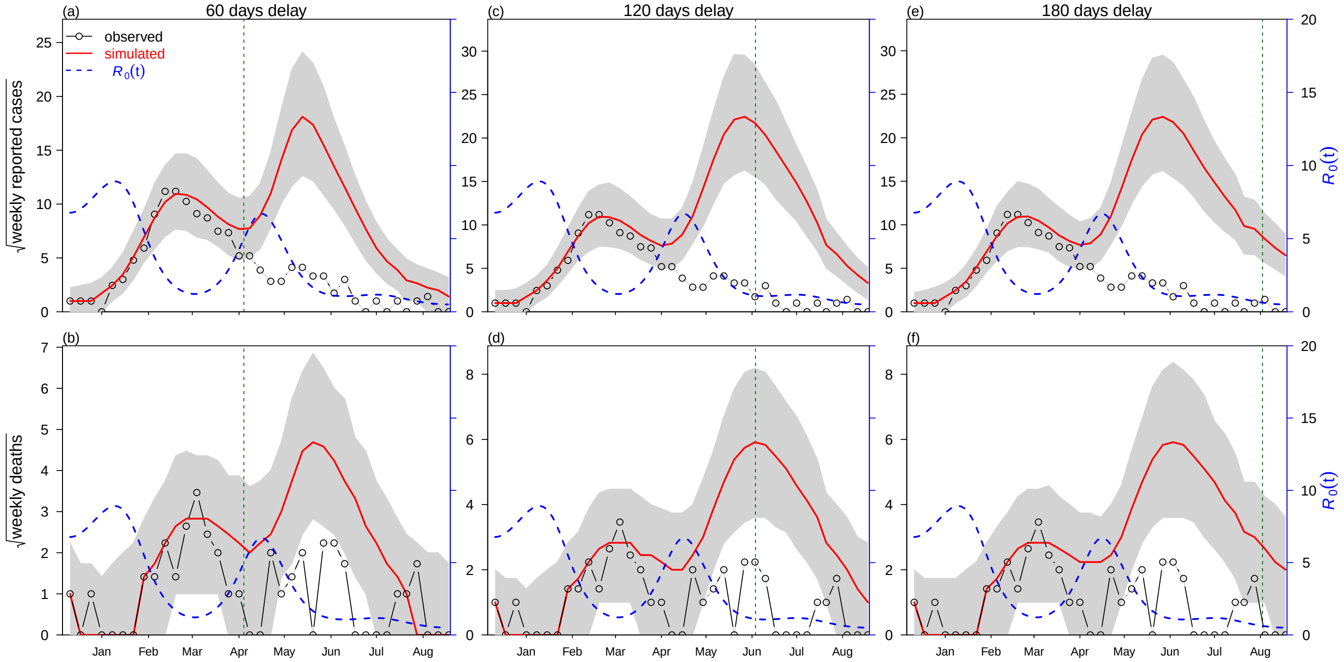

Fig S5. Simulation results for fitting CFR (weak infectivity) under delayed vaccination campaign for 60 days in panels (a,b), 120 days in panels (c, d) and 180 days in panels (e,f) delay. The case is black dotted line, model simulation median is in red and the fitted basic reproduction number, $\mathcal{R}_0$, is blue dashed line. Shaded region represents 95% range of 1,000 simulations. The vertical dashed line is the time point when the vaccination campaign started. Number of nodes, $n_m = 7$.

arise $(\delta \cdot \sigma \cdot E_h)$ but may induce a time shift of several days which is relatively small. 51

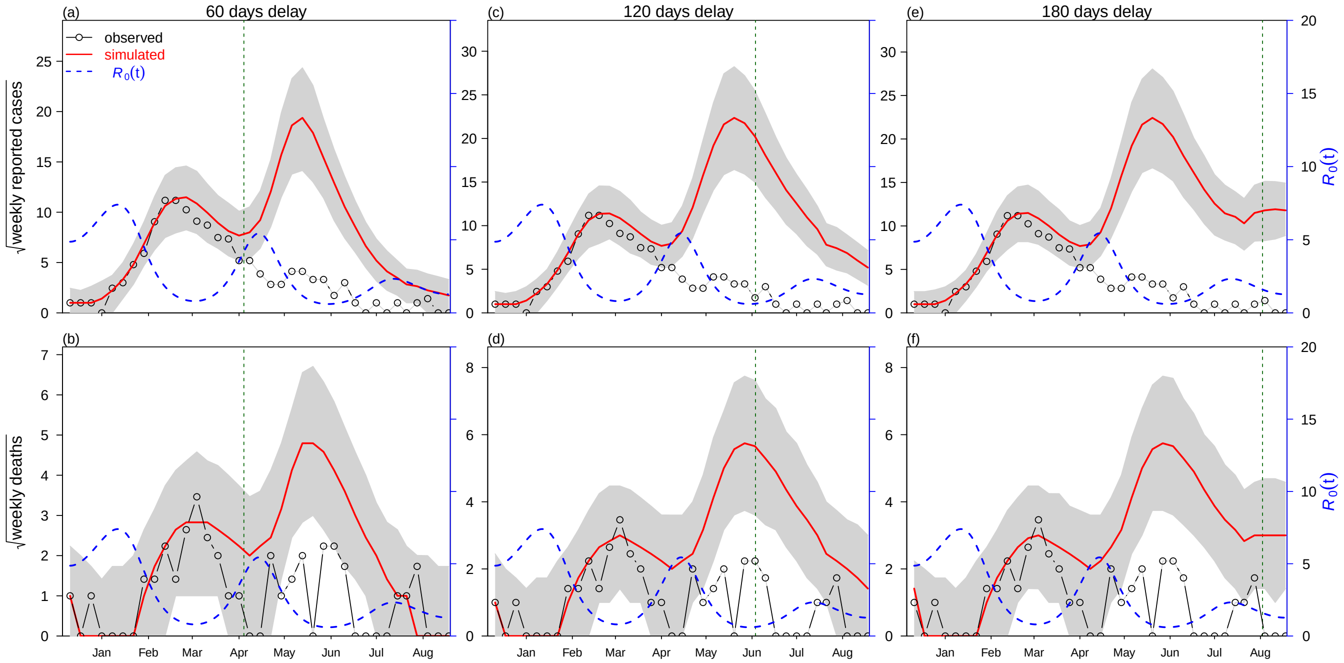

Fig S6. Simulation results for fitting CFR (strong infectivity) under delayed vaccination campaign for 60 days in panels (a,b), 120 days in panels (c, d) and 180 days in panels (e,f) delay. The case is black dotted line, model simulation median is in red and the fitted basic reproduction number, $\mathcal{R}_0$, is blue dashed line. Shaded region represents 95% range of 1,000 simulations. The vertical dashed line is the time point when the vaccination campaign started. Number of nodes, $n_m = 7$.
