# Supplementary material for: Modelling the large-scale yellow fever outbreak in Luanda, Angola, and the impact of vaccination
Source: PLoS Negl Trop Dis. 2018 Jan 16;12(1):e0006158. doi: 10.1371/journal.pntd.0006158 (PMC5798855; doi:10.1371/journal.pntd.0006158)
Supplement: S3 Text — (PDF) [file pntd.0006158.s003.pdf]

Modelling the Large-scale Yellow Fever Outbreak in Luanda, Angola, and the Impact of Vaccination

Shi Zhao[1], Lewi Stone[2,3,*], Daozhou Gao[4] & Daihai He[1,*]

1 Department of Applied Mathematics, Hong Kong Polytechnic University, Hong Kong, China
2 School of Mathematical and Geospatial Sciences, RMIT University, Melbourne, 3000, Australia
3 Biomathematics Unit, Department of Zoology, Tel Aviv University, Ramat Aviv, Israel
4 Department of Mathematics, Shanghai Normal University, Shanghai, China

* Corresponding: D.H. daihai.he@polyu.edu.hk & L.S. lewistone100@gmail.com

S3 Basic Reproduction Number

Here we derive the basic reproductive number $\mathcal{R}_0$ for the vector-host model Eqn 1. Following standard procedures [1, 2], the rate of transmission (i.e., the changing rates from infectious to non-infectious classes) is given by, $\mathcal{F}$:

$$\mathcal{F} = \begin{pmatrix} ab\frac{I_v}{N_h}S_h \\ 0 \\ 0 \\ ac\frac{\psi A_h + I_h}{N_h}S_v \\ 0 \end{pmatrix}$$

and the rate of transition (i.e., the changing rates among infectious classes), $\mathcal{V}$:

$$\mathcal{V} = \begin{pmatrix} \sigma_h E_h \\ (\delta - 1)\sigma_h E_h + \gamma_h A_h \\ -\delta\sigma_h E_h + \gamma_h I_h \\ (\sigma_v + \mu_v)E_v \\ -\sigma_v E_v + \mu_v I_v \end{pmatrix}$$

Then, we have two Jacobian matrices, of which F is the Jacobian of $\mathcal{F}$ and V is the Jacobian of $\mathcal{V}$, and we derive F and V under the disease free equilibrium [1] (DFE, by setting the proportions of susceptible classes to be 100%),

$$F = \begin{pmatrix} 0 & 0 & 0 & 0 & ab \\ 0 & 0 & 0 & 0 & 0 \\ 0 & 0 & 0 & 0 & 0 \\ 0 & ac\psi m & acm & 0 & 0 \\ 0 & 0 & 0 & 0 & 0 \end{pmatrix} \tag{S3}$$

where $F_{i,j} = \frac{\partial \mathcal{F}_i}{\partial x_j}$ with $\mathcal{F}_i$ is the i-th equation of $\mathcal{F}$ and x_j is the j-th variable of the vector $(E_h, A_h, I_h, E_v, I_v)$,

$$V = \begin{pmatrix} \sigma_h & 0 & 0 & 0 & 0 \\ (\delta-1)\sigma_h & \gamma_h & 0 & 0 & 0 \\ -\delta\sigma_h & 0 & \gamma_h & 0 & 0 \\ 0 & 0 & 0 & (\sigma_v+\mu_v) & 0 \\ 0 & 0 & 0 & -\sigma_v & \mu_v \end{pmatrix} \tag{S4}$$

where $V_{i,j} = \frac{\partial \mathcal{V}_i}{\partial x_j}$ with $\mathcal{V}_i$ is the i-th equation of $\mathcal{V}$ and x_j is the j-th variable of the vector $(E_h,\, A_h,\, I_h,\, E_v,\, I_v)$. Then, we have the next generation matrix of our model,

$$FV^{-1} = \begin{pmatrix} 0 & 0 & 0 & \frac{ab\sigma_v}{\mu_v(\sigma_v+\mu_v)} & \frac{ab}{\mu_v} \\ 0 & 0 & 0 & 0 & 0 \\ 0 & 0 & 0 & 0 & 0 \\ \frac{acm}{\gamma_h}\cdot(\psi+\delta-\psi\delta) & \frac{acm}{\gamma_h}\cdot\psi & \frac{acm}{\gamma_h} & 0 & 0 \\ 0 & 0 & 0 & 0 & 0 \end{pmatrix} \tag{S5}$$

Since $\mathcal{R}_0$ is the spectral radius of FV^{-1} [1], the basic reproduction number is

$$\mathcal{R}_0 = \sqrt{(\psi+\delta-\psi\delta)\cdot\frac{a^2bcm}{\gamma_h}\cdot\frac{\sigma_v}{\mu_v(\sigma_v+\mu_v)}} \tag{S6}$$

where m is the ratio of mosquito-to-human population.

References

1. van den Driessche P, Watmough J. Reproduction numbers and sub-threshold endemic equilibria for compartmental models of disease transmission. Math Biosci. 2002;180:29-48.
2. Brauer F, Castillo-Chavez C, Mubayi A, Towers S. Some models for epidemics of vector-transmitted diseases. Infectious Disease Modelling. 2016;1:79-87.
