# Supplementary material for: Modelling the large-scale yellow fever outbreak in Luanda, Angola, and the impact of vaccination
Source: PLoS Negl Trop Dis. 2018 Jan 16;12(1):e0006158. doi: 10.1371/journal.pntd.0006158 (PMC5798855; doi:10.1371/journal.pntd.0006158)
Supplement: S4 Text — (PDF) [file pntd.0006158.s004.pdf]

Modelling the Large-scale Yellow Fever Outbreak in Luanda, Angola, and the Impact of Vaccination

Shi Zhao[1], Lewi Stone[2,3,*], Daozhou Gao[4] & Daihai He[1,*]

1 Department of Applied Mathematics, Hong Kong Polytechnic University, Hong Kong, China
2 School of Mathematical and Geospatial Sciences, RMIT University, Melbourne, 3000, Australia
3 Biomathematics Unit, Department of Zoology, Tel Aviv University, Ramat Aviv, Israel
4 Department of Mathematics, Shanghai Normal University, Shanghai, China

* Corresponding: D.H. daihai.he@polyu.edu.hk & L.S. lewistone100@gmail.com

S4 Estimating the Confidence Interval of $\mathcal{R}_0(t)$

Figs. S4 and S5 show the 95% confidence interval (CI) for the estimated $\mathcal{R}_0$, obtained by calculating the 95% CI of $m(t)$, which is obtained from calculating the profile maximum log likelihood of the model as a function of value of each node of $m(t)$. The width of the CI became very wide in the last 2/7 (i.e., 28.57%) of the study period because both case numbers and the number of deaths became relatively small and noisy.

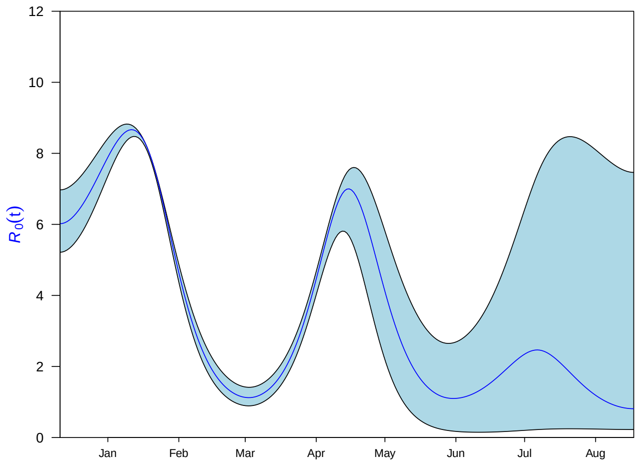

Fig S4. The Confidence Interval (C.I.) estimation plot of $\mathcal{R}_0$ under scenario 1.

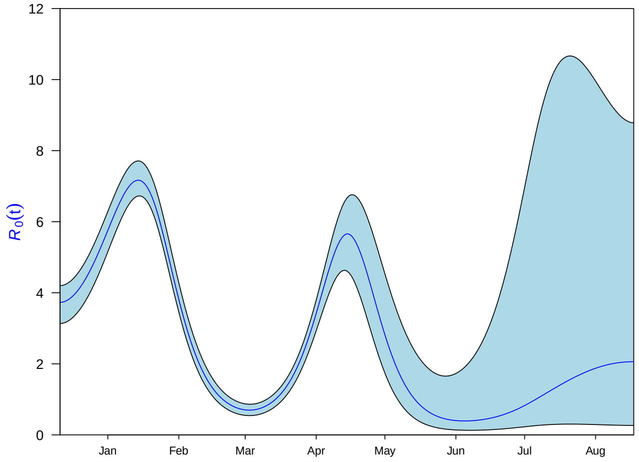

Fig S5. The Confidence Interval (C.I.) estimation plot of $\mathcal{R}_0$ under scenario 2.
