# Supplementary material for: Modelling the large-scale yellow fever outbreak in Luanda, Angola, and the impact of vaccination
Source: PLoS Negl Trop Dis. 2018 Jan 16;12(1):e0006158. doi: 10.1371/journal.pntd.0006158 (PMC5798855; doi:10.1371/journal.pntd.0006158)
Supplement: S5 Text — (PDF) [file pntd.0006158.s005.pdf]

Modelling the Large-scale Yellow Fever Outbreak in Luanda, Angola, and the Impact of Vaccination

Shi Zhao[1], Lewi Stone[2,3,*], Daozhou Gao[4] & Daihai He[1,*]

1 Department of Applied Mathematics, Hong Kong Polytechnic University, Hong Kong, China
2 School of Mathematical and Geospatial Sciences, RMIT University, Melbourne, 3000, Australia
3 Biomathematics Unit, Department of Zoology, Tel Aviv University, Ramat Aviv, Israel
4 Department of Mathematics, Shanghai Normal University, Shanghai, China

* Corresponding: D.H. daihai.he@polyu.edu.hk & L.S. lewistone100@gmail.com

S5 Underlying Oscillation in Basic Reproductive Number

S5.1 Import of New Susceptibles

In order to explain the multiple waves in $\mathcal{R}_0$, one might speculate that there were additional inflows of new susceptibles in the later stage of epidemic. But we show that even if there are, the estimated $\mathcal{R}_0$ still exhibits features of multiple waves. We examined the addition to the system of a continuous inflow (daily 10,000 after 04/09) or a square-wave inflow (daily 20,000 for the period 03/30-04/29 and 06/08-07/07) of imported host susceptibles (as well as cross border infections from confirmed cases outside Luanda province). These input of new susceptibles resulted in little difference to the baseline scenario (see Figs. S5. Scenario 1's parameters were used here).

S5.2 Modelling Human reaction to mortality

We have seen that the reproductive number $\mathcal{R}_0(t)$ exhibits multiple waves. An examination of the weekly YF mortality reveals possibly related oscillations. High weekly mortality corresponds to high transmission rate. Given that in practice, the vector control (mosquito-fogging) was case-driven (fogging was implemented in localities where cases and mortality were observed), it is not totally unreasonable to link mortality to the mosquito-abundance and the transmission rate (see a discussion of this in section S9). In this section, we use a simple model simulation to demonstrate this possibility.

We replace the cubic spline function (with 7 nodes) for $m(t)$ (Eqn. 4) with a simple function based on the YF mortality and obtain almost identical results. We set:

$$m(t) = m_{\text{base}} + k \cdot \exp\Big[-D_h(t - t_{\text{lag}})\Big] \tag{S5}$$

Here m_{base} is a constant term, k is a parameter controlling the strength of the death-induced human reaction, $D_h(t)$ is the yellow fever deaths of week t and t_{lag} is the lag time for the population response in reaction to mortality levels. The fitting results for this simple human behavior model are shown in Fig. S6, with $t_{\text{lag}} = 1$ week fixed. The estimated $m_{\text{base}} = 0.4951$, $k = 19.1125$ for the weak infectivity scenario (scenario 1), and $m_{\text{base}} = 0.2213$, $k = 7.8516$ for the strong infectivity scenario (scenario 2). We

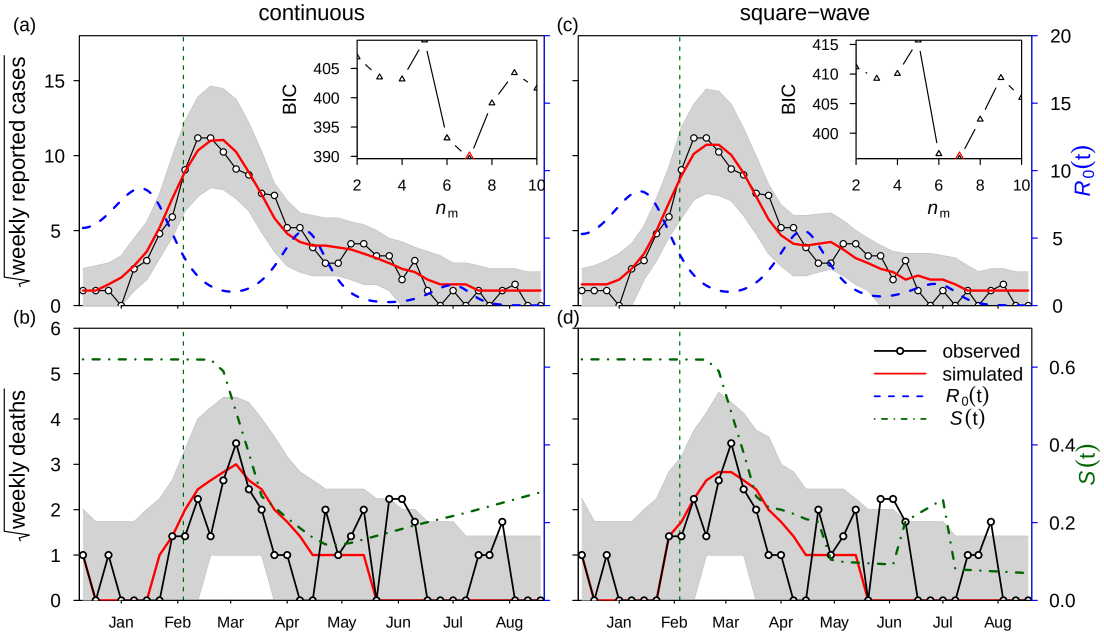

Fig S5. Fitting results for model including a continuous (a,b) or square-wave(c,d) inflow of new susceptibles. Weekly reported cases (a,c) and deaths cases (b,d) in Luanda province, Angola. Black line with circles denotes reported cases, and red line denotes model simulation median, blue dashed line denotes the fitted basic reproduction number, $\mathcal{R}_0$, and green dashed line shows the calculated host susceptible proportion, $S(t)$. Shaded region represents 95% bound of 1000 simulations. The vertical dashed line indicates the starting date of the vaccination campaign. Inset panel shows the BIC as a function of the number of nodes (n_m). The lowest BIC is attained at $n_m = 7$, which is used in the main panel.

see that a simple behavior model, achieved very similar fits as our original model in Fig. 3. Thus we illustrated that death-driven oscillation in $\mathcal{R}_0$ is possible.

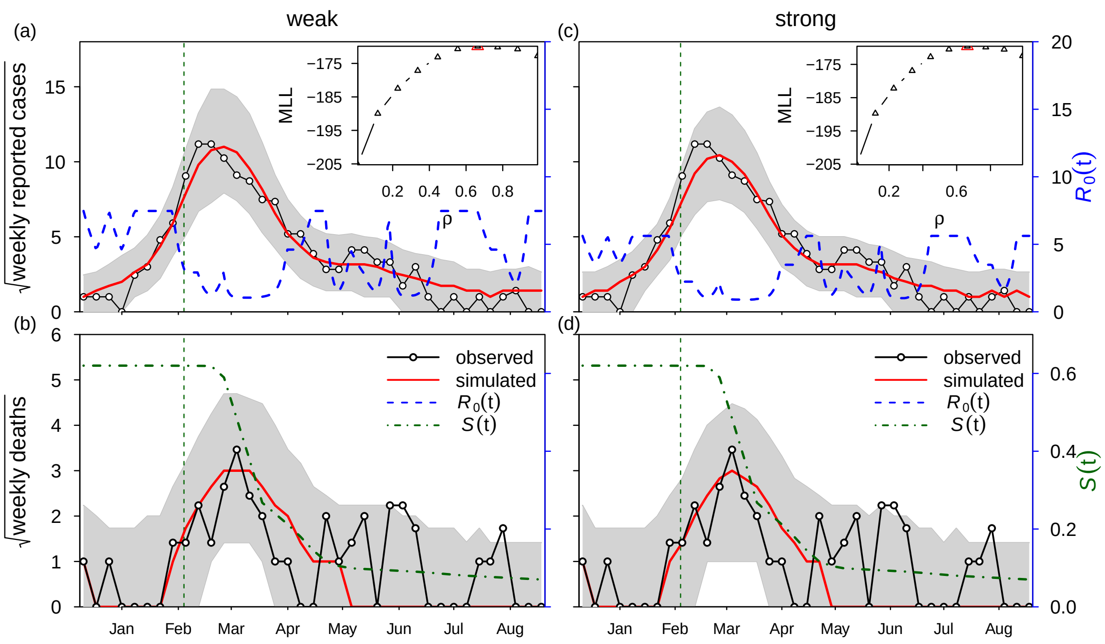

Fig S6. Fitting results implementing humans reaction to mortality (with a time lag of one week, $t_{\mathrm{lag}} = 1$), scenario 1 (a,b) and scenario 2 (c,d). Black line with circles denotes reported cases, and red line denotes model simulation median, blue dashed line denotes the fitted basic reproduction number, $\mathcal{R}_0$, and green dashed line shows the calculated host susceptible proportion, $S(t)$. Shaded region represents 95% bound of 1000 simulations. The vertical dashed line indicates the start date of the vaccination campaign. Inset panel shows the MLL as a function of the mild YF infections counting ratio (ρ). The highest MLL is used in the main panel.
