# Supplementary material for: Modelling the large-scale yellow fever outbreak in Luanda, Angola, and the impact of vaccination
Source: PLoS Negl Trop Dis. 2018 Jan 16;12(1):e0006158. doi: 10.1371/journal.pntd.0006158 (PMC5798855; doi:10.1371/journal.pntd.0006158)
Supplement: S6 Text — (PDF) [file pntd.0006158.s006.pdf]

Modelling the Large-scale Yellow Fever Outbreak in Luanda, Angola, and the Impact of Vaccination

Shi Zhao[1], Lewi Stone[2,3,*], Daozhou Gao[4] & Daihai He[1,*]

1 Department of Applied Mathematics, Hong Kong Polytechnic University, Hong Kong, China
2 School of Mathematical and Geospatial Sciences, RMIT University, Melbourne, 3000, Australia
3 Biomathematics Unit, Department of Zoology, Tel Aviv University, Ramat Aviv, Israel
4 Department of Mathematics, Shanghai Normal University, Shanghai, China

* Corresponding: D.H. `daihai.he@polyu.edu.hk` & L.S. `lewistone100@gmail.com`

S6 Impacts of Temperature and Rainfall

Climatic data for Luanda Angola were obtained from Weather Underground Organization (`https://www.wunderground.com`). Fig. S6 shows the weekly reported cases and deaths and daily mean air temperature and rainfall (in mm). Over the study period, the air temperature decreased and the rainfall concentrated in a few months. There is no evident association between temperature/rainfall and reported cases/deaths by inspection, except that the rainfall in April/May might be associated with the second wave in reported cases.

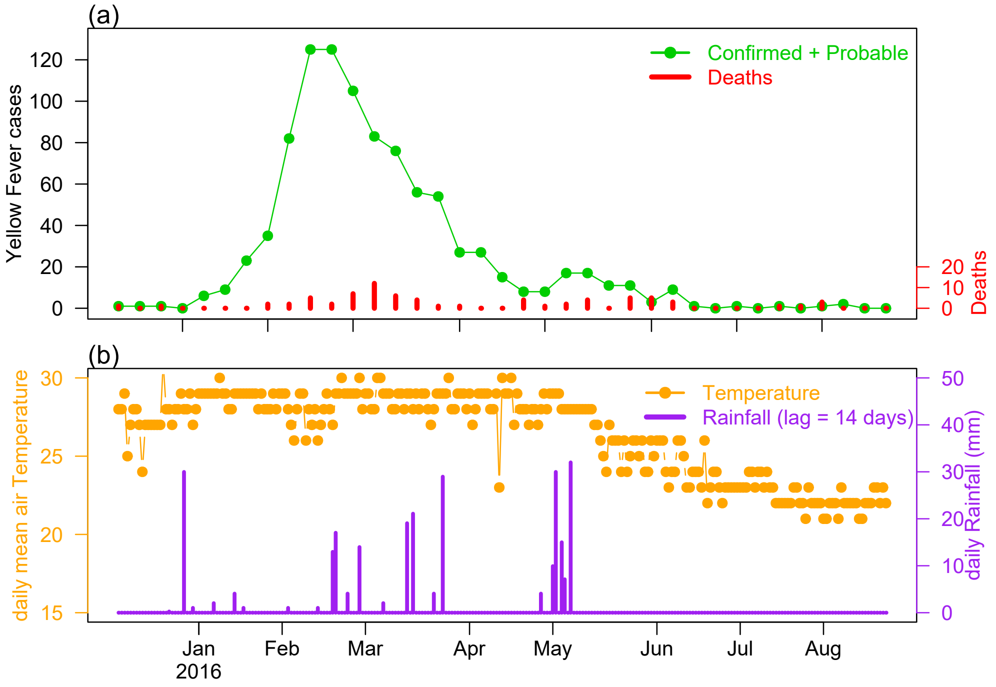

Fig S6. Weekly reported cases and deaths in Luanda Angola (a) and daily mean air temperature and rainfall (mm) (b).

Daily temperature and rainfall are obtained for Luanda airport, Angola. We assume the mosquito human population size ratio is:

$$m(t) = m_{\text{base}}(t) + \xi_1 \cdot [\text{TEMP}(t) - \min\{\text{TEMP}\}] + \xi_2 \cdot \text{RAIN}(t - t_d) \qquad \text{(S6)}$$

where $m_{\text{base}}(t)$ is an exponential cubic spline function with $n_m = 7$ nodes, TEMP (with minimum value, min{TEMP}) and RAIN are daily temperature (in °C) and rainfall (in mm) respectively, and ξ_1 and ξ_2 are parameters to be estimated. A time delay $t_d = 14$ days is considered for rainfall. In this simulation, we integrated the model with a time-step-size of 1 day. If temperature or rainfall played a significant role, we expect to detect a non-zero ξ_1 or ξ_2 with a significantly improved fitting. But the fitting is not improved significantly (see Fig. S7) with an estimated $\xi_1 = 0.05$ and $\xi_2 = 0.2$.

From the MLL in the inset to Fig. S7 (panel c) we can conclude that temperature had minor non-significant effect in the long-run, while the effect of rainfall was significant. Quite likely rainfall had major impact on mosquito abundances (i.e., parameter $m(t)$) in Mar 2016, due to the strong El Niño flood events [1]. While the rainfall parameter $\xi_2 = 0.2$ is significant, the true effects of rainfall may be hidden in the baseline cubic fit $m_{\text{base}}(t)$, and thus hard to untangle. Nevertheless, the increased March 2016 rainfall period may be at least partly responsible for the second peak in $\mathcal{R}_0(t)$.

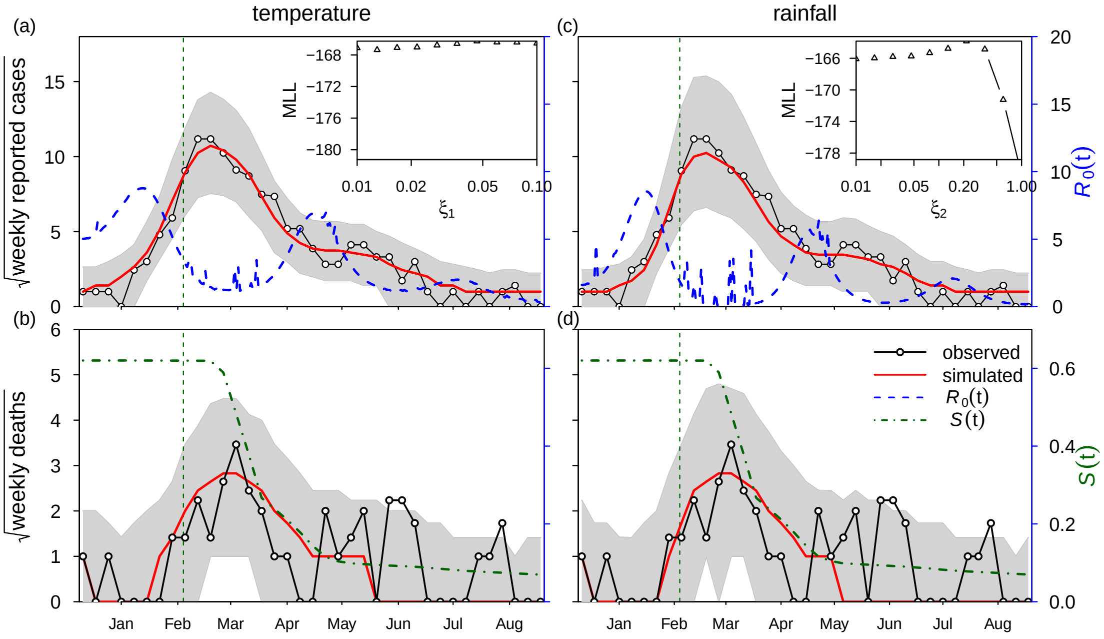

Fig S7. Fitting results with climate factors: local temperature (a,b) and rainfall (c,d). Inset panel shows the profile MLL as a function of ξ_1. Black line with circles denotes reported cases, and red line denotes model simulation median, blue dashed line denotes the fitted basic reproduction number, $\mathcal{R}_0$. Inset panel shows the MLL as a function of the temperature parameter (ξ_1). The highest MLL is used in the main panel.

The results of correlation tests between climatic time series and YF time series (see Table S6) shows that none of the correlations are significant.

References

1. http://www.unocha.org/legacy/el-nino-southern-africa

Table S6. Correlation results

Correlations	weekly cases	weekly deaths
weekly temperature	−0.1756 (p-value=0.30)	−0.2451 (p-value=0.14)
weekly rainfall	−0.1659 (p-value=0.33)	−0.0740 (p-value=0.66)
weekly rainfall (lagged 14 days)	−0.0290 (p-value=0.86)	−0.1058 (p-value=0.53)
