# Supplementary material for: Modelling the large-scale yellow fever outbreak in Luanda, Angola, and the impact of vaccination
Source: PLoS Negl Trop Dis. 2018 Jan 16;12(1):e0006158. doi: 10.1371/journal.pntd.0006158 (PMC5798855; doi:10.1371/journal.pntd.0006158)
Supplement: S7 Text — (PDF) [file pntd.0006158.s007.pdf]

Modelling the Large-scale Yellow Fever Outbreak in Luanda, Angola, and the Impact of Vaccination

Shi Zhao[1], Lewi Stone[2,3,*], Daozhou Gao[4] & Daihai He[1,*]

1 Department of Applied Mathematics, Hong Kong Polytechnic University, Hong Kong, China
2 School of Mathematical and Geospatial Sciences, RMIT University, Melbourne, 3000, Australia
3 Biomathematics Unit, Department of Zoology, Tel Aviv University, Ramat Aviv, Israel
4 Department of Mathematics, Shanghai Normal University, Shanghai, China

* Corresponding: D.H. daihai.he@polyu.edu.hk & L.S. lewistone100@gmail.com

S7 Assuming deaths were under-reported (CFR=15%)

To further test the robustness of our conclusion, we assume that deaths were under-reported. We assume the "actual" YF-related deaths were double the confirmed deaths, so that the "actual" death toll is $2 \times 73 = 146$. We then set the CFR = 15%, which is in line with previous studies in Africa (see "*the historic estimate of 200,000 cases and 30,000 deaths annually, which was based on serological survey data obtained from children in Nigeria between 1945 and 1971*" in [1]). Results are summarized in Fig. S7 and Tables S7 and S8. Our key conclusions still hold, namely the deaths prevented are approximately 5,6-fold times the reported "actual" death toll.

Table S7. Impacts of vaccination campaign delay under weak infectivity scenario and CFR = 15%**.**

Scenario	Total reported cases	Total deaths
Observed	941	146
Baseline model	938 [486 , 1669]	149 [76 , 264]
60 days delay	3425 [1736 , 6168]	538 [263 , 962]
120 days delay	5906 [2829 , 10529]	917 [450 , 1678]
180 days delay	6266 [3104 , 11220]	957 [481 , 1684]

Table S8. Impacts of vaccination campaign delay under strong infectivity scenario and CFR = 15%**.**

Scenario	Total reported cases	Total deaths
Observed	941	146
Baseline model	896 [431 , 1611]	148 [69 , 271]
60 days delay	3325 [1579 , 5846]	538 [250 , 984]
120 days delay	5508 [2676 , 9995]	910 [443 , 1618]
180 days delay	6146 [2932 , 11034]	978 [450 , 1779]

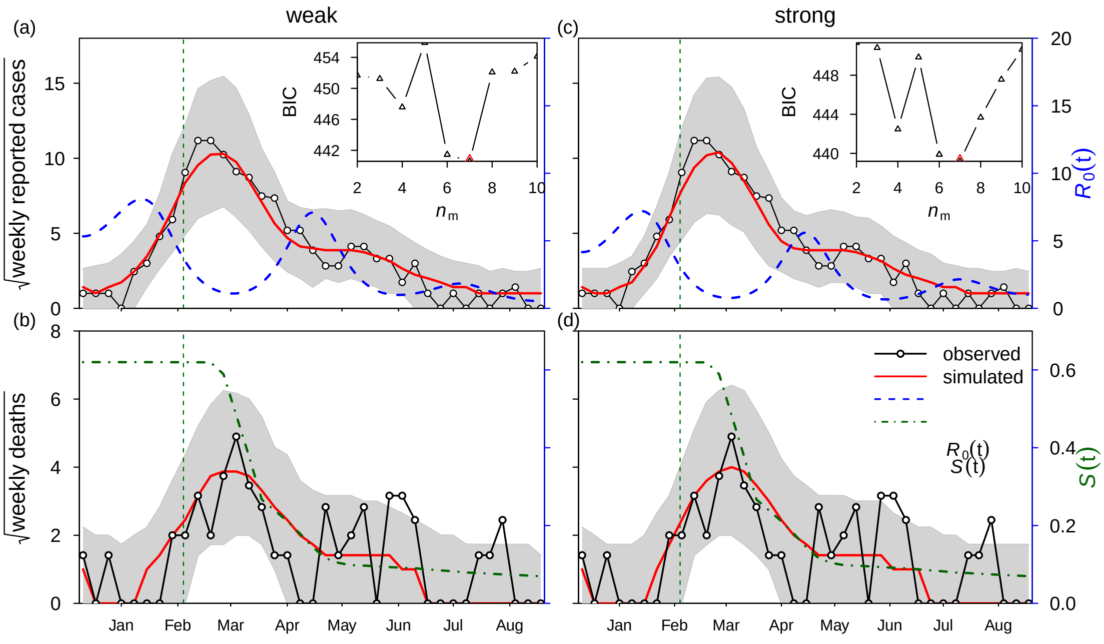

Fig S7. Fitting a model with mocked deaths data and CFR = 15%

References

1. Garske T, Van Kerkhove MD, Yactayo S, Ronveaux O, Lewis RF, Staples JE, ... & Yellow Fever Expert Committee. Yellow Fever in Africa: estimating the burden of disease and impact of mass vaccination from outbreak and serological data. PLoS Med. 2014;11(5), e1001638.
