# Supplementary material for: Modelling the large-scale yellow fever outbreak in Luanda, Angola, and the impact of vaccination
Source: PLoS Negl Trop Dis. 2018 Jan 16;12(1):e0006158. doi: 10.1371/journal.pntd.0006158 (PMC5798855; doi:10.1371/journal.pntd.0006158)
Supplement: S8 Text — (PDF) [file pntd.0006158.s008.pdf]

Modelling the Large-scale Yellow Fever Outbreak in Luanda, Angola, and the Impact of Vaccination

Shi Zhao[1], Lewi Stone[2,3,*], Daozhou Gao[4] & Daihai He[1,*]

1 Department of Applied Mathematics, Hong Kong Polytechnic University, Hong Kong, China
2 School of Mathematical and Geospatial Sciences, RMIT University, Melbourne, 3000, Australia
3 Biomathematics Unit, Department of Zoology, Tel Aviv University, Ramat Aviv, Israel
4 Department of Mathematics, Shanghai Normal University, Shanghai, China

* Corresponding: D.H. daihai.he@polyu.edu.hk & L.S. lewistone100@gmail.com

S8 Impact of vector control and possible impact of climate

We discuss how controlling mosquito eradication programs could also substantially influenced mosquito populations. Note that the government programs on vector control are often driven by reported cases and deaths. When deaths were reported from a local community, the vector control team was sent on location for fogging the streets with chemical spray as a means of destroying mosquitos. Other factors controlling the vector population might also have an impact. Dr. Moreira has outlined that the WHO's response was organized into five different stages and that the reaction and support of the public was varied at different times from strong resistance to cooperation [1].

Dr. Moreira states: "The government set up a task force to lead the response and launched a five-part plan. The first part was active surveillance. We reinforced laboratory capacity to allow for early detection and notification of new cases."

"The outbreak coincided with unusually heavy rains and a severe El Niño weather pattern. We are also suffering from an economic crisis and poor sanitary conditions. All these factors created a fertile environment for an increase in the mosquito population. The outbreak reached its peak in February and has been declining since. We have much more vaccine now [in September 2016] than we had earlier in the epidemic. The response interventions are involving communities successfully. The dry season arrived in May [2016] and since then the mosquito population has diminished."

The second cycle The rainy season is between November - May but the most accumulation of rain in Luanda occurs in March - April [2].

S8.1 Details of control program

As discussed, major control efforts aimed to control the mosquito vector population *Aedes aegypti* (which transmits the YF virus) were ongoing during the observation period. The Mission Report of the European Centre for Disease Prevention and Control "Assessing the yellow fever outbreak in Angola" [3] outlines the control efforts.

•Suspected case: inspection of the case's residence and the surrounding homes within a 200m radius; calculation of larval and adult infestation levels

•Confirmed case: indoor fogging of the case's residence and surrounding homes within a 200m radius

•Neighbourhoods with a high infestation level: truck-fogging with cypermethrine, door-to-door visits to inspect potential breeding sites, Bti application, and informing household members about control measures for *Aedes* control

•In remote areas: fogging of residence and surroundings when a resident returned from Luanda

•Information/education/communication messages on household control measures distributed through various channels to achieve community actions

•Bti or cypermethrine are not always available because purchase depends on the municipality's priority settings. Application is hampered by the high cost of fuel which needs to be mixed with the adulticide. The municipal vector control teams are often not aware of the main *Aedes* breeding sites or geographical high-risk pockets. *Aedes* infestation levels are reaching a Breteau index of 50 to 103 in some geographical pockets while in other areas no *Aedes* mosquitoes were present. Only *Aedes aegypti* has been identified, with neither *Aedes albopictus* nor *Aedes africanus* found. All in all, it remains difficult to determine the geographical distribution of the vector, the corresponding infestation levels, and the entomological risk for local transmission.

Vector control/entomology during the epidemic: As confirmation of cases is often only available after several days, it is essential to ensure that fogging is performed as soon as a suspected case is detected; waiting for case confirmation results in losing valuable time.

The vector control measure was driven by reported cases (see the above), and also by reported deaths, but also under limited resources. We therefore make the not unreasonable assumption that $\mathcal{R}_0$ may be driven by reported deaths, as has been done in the past in a number of studies [4, 5]. (It was thought that the CFR of YF could be as high as 47%. Thus it should not be surprising if there were panic at the initial stage of the outbreak.)

Dr. Moreira's account of vector control and vaccination

Dr. Moreira has outlined that the WHO's response was organized into five different stages and that the reaction and support of the public was varied at different times from strong resistance to cooperation.

In [1], the WHO medical epidemiologist, Dr. Moreira states: "The government set up a task force to lead the response and launched a five-part plan. The first part was active surveillance. We reinforced laboratory capacity to allow for early detection and notification of new cases. The second part was case management. With the help of Médecins Sans Frontières, we developed case management guidelines and distributed these to different provinces because there is no specific treatment for yellow fever. We also provided health workers with a flowchart indicating six health facilities where people with severe disease should be referred. By February, about 30% of some 300 people with confirmed yellow fever had died. After implementing our plan, case–fatality dropped to 11%. The third part was mass vaccination, and the fourth was integrated vector control measures to lower the density of the *Aedes aegypti* mosquito that carries yellow fever, as well as dengue, chikungunya and Zika viruses. The fifth part of the strategy was risk communication and social mobilization."

"We began working in Viana, a marketplace attracting people from all over Angola. It's difficult to control the movement of people and the area has major sanitation problems. Mosquito density was high, so we carried out a mass distribution of larvicide and a social mobilization campaign to explain to people how to use it. We also did indoor and outdoor spraying with insecticides. Initially we faced resistance: some people kept the larvicide at home and did not use it. So we asked community leaders to help us persuade people to join the campaign. As a result of these vector control measures, the mosquito density level fell substantially."

"We have been conducting fixed post and mobile vaccination campaigns in 73 districts. By 7 September about 16 million individuals, 65% of the Angolan population, had been vaccinated – most of them in reactive campaigns, where local transmission was confirmed. In addition, 3 million of these individuals were vaccinated in a preventive campaign in August and September. "

References

1. Moreira R. Reining in Angola's yellow fever outbreak. Bulletin of the World Health Organization 2016; 94:716-717.
2. del Carmen Marquetti Fernandez M, Flores YH, & Nuviola DL. Spatial Distribution and Mainly Breeding Sites of Aedesaegypti (Diptera:Culicidae) in Luanda, Angola. Ann Community Med Pract. 2017; 3(1), 1017.
3. European Centre for Disease Prevention and Control. Mission report: Yellow fever outbreak in Angola. 2016
4. Bootsma MC & Ferguson NM. The effect of public health measures on the 1918 influenza pandemic in US cities. Proc Natl Acad Sci U S A. 2007;104(18), 7588-7593.
5. He D, Dushoff J, Day T, Ma J, & Earn DJ. Inferring the causes of the three waves of the 1918 influenza pandemic in England and Wales. Proc R Soc Lond B Biol Sci. 2013; 280(1766), 20131345.
